# Pulcherrimin protects *Bacillus subtilis* against oxidative stress during biofilm development

**DOI:** 10.1038/s41522-023-00418-z

**Published:** 2023-07-19

**Authors:** Leticia Lima Angelini, Renato Augusto Corrêa dos Santos, Gabriel Fox, Srinand Paruthiyil, Kevin Gozzi, Moshe Shemesh, Yunrong Chai

**Affiliations:** 1 Department of Biology, Northeastern University, Boston, MA 02115 USA; 2 Centro de Energia Nuclear na Agricultura, Universidade de São Paulo, Piracicaba, SP 13400-970 Brazil; 3 Department of Food Science, Agricultural Research Organization The Volcani Institute, Derech Hamacabim, POB 15159, Rishon LeZion, 7528809 Israel; 4 Present Address: Medical Scientist Training Program (MSTP), Washington University School of Medicine, 660 S Euclid Ave, St. Louis, MO 63110 USA; 5 Present Address: The Rowland Institute at Harvard, 100 Edwin H. Land Blvd., Cambridge, MA 02142 USA

**Keywords:** Biofilms, Bacteriology

## Abstract

Pulcherrimin is an iron-binding reddish pigment produced by various bacterial and yeast species. In the soil bacterium *Bacillus subtilis*, this pigment is synthesized intracellularly as the colorless pulcherriminic acid by using two molecules of tRNA-charged leucine as the substrate; pulcherriminic acid molecules are then secreted and bind to ferric iron extracellularly to form the red-colored pigment pulcherrimin. The biological importance of pulcherrimin is not well understood. A previous study showed that secretion of pulcherrimin caused iron depletion in the surroundings and growth arrest on cells located at the edge of a *B. subtilis* colony biofilm. In this study, we identified that pulcherrimin is primarily produced under biofilm conditions and provides protection to cells in the biofilm against oxidative stress. We presented molecular evidence on how pulcherrimin lowers the level of reactive oxygen species (ROS) and alleviates oxidative stress and DNA damage caused by ROS accumulation in a mature biofilm. We also performed global transcriptome profiling to identify differentially expressed genes in the pulcherrimin-deficient mutant compared with the wild type, and further characterized the regulation of genes by pulcherrimin that are related to iron homeostasis, DNA damage response (DDR), and oxidative stress response. Based on our findings, we propose pulcherrimin as an important antioxidant that modulates *B. subtilis* biofilm development.

## Introduction

Biofilms are architecturally complex bacterial communal structures. Biofilms consist of a multitude of cells embedded in a self-produced extracellular matrix mainly composed of exopolysaccharides, proteins, and sometimes DNA^1,2^. The Gram-positive soil bacterium *Bacillus subtilis* is a well-studied species with robust biofilm-forming capabilities, which has made it an ideal model for biofilm research^2–4^. In *B. subtilis*, matrix production and secretion allow cells to attach to solid surfaces as a colony biofilm, or to grow at the air-liquid interface in a pellicle biofilm in the laboratory^1,5^. In natural environments, the biofilm matrix is shown to play important roles in facilitating attachment of *B. subtilis* cells to the surface of plant roots or even fungal hyphae^6–8^. Within the biofilm, cells are divided into subpopulations, and each of them is responsible for performing a different task, such as matrix production, DNA uptake (competency), or synthesis of antibiotics and other secondary metabolites^9,10^.

Approximately 5% of the *B. subtilis* genome is dedicated to the production of secondary metabolites^11^. Some of these are ribosomally synthesized, while others are non-ribosomal peptides (NRPs)^12^. One important category of secondary metabolites produced by *B. subtilis* are siderophores, which are iron chelators with the main role of scavenging iron from the environment^13,14^. Iron is an essential trace element whose bioavailability is often scarce in aerobic environments at neutral pH^14,15^. Secreted siderophores are able to bind iron, increase bioavailability of iron, and facilitate iron acquisition. Bacteria have also developed molecular mechanisms that regulate iron homeostasis and ensure optimal intracellular iron levels. In *B. subtilis*, a crucial regulator of iron homeostasis is the ferric uptake regulator (Fur). In conditions where iron is scarce, Fur derepresses iron acquisition systems, including bacillibactin, the most common catecholic siderophore produced by *B. subtilis*^16,17^. Bacillibactin is a high-affinity iron-binding NRP, synthesized through a multistep process carried out by enzymes encoded in the *dhbACEBF* operon^18,19^. Upon iron binding, bacillibactin forms orange-colored soluble iron complexes, which can be taken up by the cells through specific transporters^20^.

The cyclic dipeptide pulcherrimin produced by *B. subtilis* is also an NRP and a secondary metabolite. The biosynthesis pathway of pulcherrimin involves the activity of two key enzymes; a cyclodipeptide synthase YvmC, which catalyzes the formation of cyclic-di-leucine from two leucine-charged tRNA molecules, and a cytochrome P450 oxidase CypX, which oxidizes the cyclic dipeptide, yielding pulcherriminic acid. Once this molecule is secreted to the extracellular environment, it chelates ferric iron (Fe^+3^), turning itself into the reddish pigment pulcherrimin^21–23^. The biosynthetic operon of pulcherrimin, *yvmC-cypX*, is regulated by AbrB, a transition-state transcriptional regulator known to repress the synthesis of secondary metabolites during the exponential phase^23–25^. In addition, PchR is a negative regulator of pulcherrimin biosynthesis. This MarR-like transcription repressor directly binds to the promoter of the *yvmC-cypX* operon and represses expression of these biosynthetic genes^26^. The biological importance of pulcherrimin is still not well understood. One previous study in the yeast *Kluyveromyces lactis* demonstrated that pulcherrimin functions as a siderophore that is taken up by the cells through a dedicated transporter^27^. Another study in *B. subtilis* showed that secretion of pulcherrimin can cause growth arrest and inhibit the expansion of colony biofilms through localized iron depletion^21^. In *B. subtilis*, no evidence was provided whether cells use pulcherrimin as iron acquisition-like siderophores even though pulcherrimin is a known iron chelator^21^.

Even though iron plays an essential role in growth and maintenance of proper cellular functions, this metal can be harmful to cells when present in excess intracellularly because it can trigger the Fenton reaction^28^. Intracellular Fe-S clusters can be damaged by reactive oxygen species (ROS) generated during cell metabolism, such as superoxide (O_2_^-^) or peroxide (H_2_O_2_), leading to the release of free ferrous ions (Fe^+2^). Through the Fenton reaction, the liberated Fe^+2^ can then catalyze H_2_O_2_ to produce hydroxyl radicals (OH)^29^. These are the most harmful reactive species of oxygen that can damage a variety of biological molecules such as lipids, proteins, and DNA^30–32^. Thus, iron homeostasis is crucial for maintaining the proper range of intracellular iron levels in bacteria to avoid damage of biomolecules. Iron also supports other functions beyond being a critical growth element. In *B. subtilis*, excess amounts of iron (hundred times more than what is needed for normal growth) need to be supplemented in the media to promote robust biofilm formation. It was further demonstrated that those excess iron ions were mostly associated with the biofilm matrix, presumably playing a role in extracellular electron transfer, electric signaling, etc^33,34^. However, it was unclear how *B. subtilis* cells in the biofilm avoid excessive iron-triggered toxicity and in what form those iron ions are present.

DNA damage seems prevalent during *B. subtilis* biofilm development, especially in mature biofilms^35^. This is probably due to accumulation of ROS as a byproduct of cell metabolism and the slow diffusion of ROS in the matrix-embedded biofilm^36^. DNA damage induces the SOS response for DNA repair via proteins RecA and LexA^37–39^. In the presence of DNA damage, RecA binds to single-stranded DNA, forming the RecA nucleoprotein filament (RecA*). RecA* then interacts with LexA to promote its autocleavage^40^. LexA is the master repressor of DNA damage response genes; when LexA undergoes autoproteolysis, derepression of DDR genes happens and the SOS response is activated^41–44^. DNA damage broadly affects bacterial biofilms in different ways. In *B. subtilis*, previous work found that biofilm-producing cells that have previously expressed matrix genes will transition to DDR gene expression after DNA damage is induced by ROS^35^.

In this study, we were interested in learning about novel functions of pulcherrimin during *B. subtilis* biofilm development. We present evidence and propose that pulcherrimin is an antioxidant produced with the goal of buffering iron ions from the extracellular environment in order to prevent DNA damage through generation of ROS.

## Results

### Biofilm conditions promote strong production of pulcherrimin, an iron chelator in *B. subtilis*

**Fig. 1:**
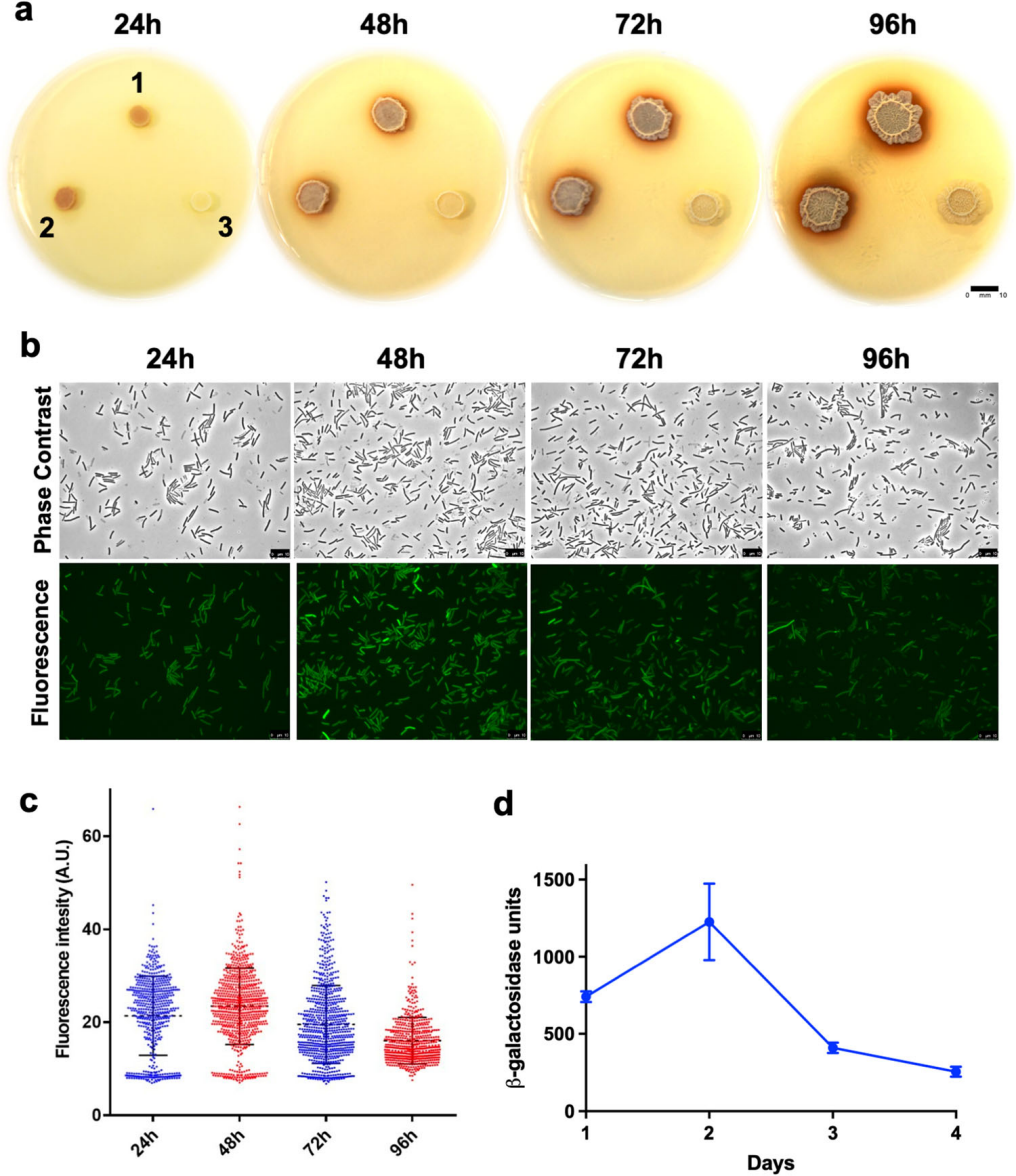
Strong production of pulcherrimin and activities of its biosynthetic genes during *B. subtilis* biofilm development. **a** Colony biofilms of the *B. subtilis* wild-type strain (3610, 1), the complementation strain (LA33, 2), and the pulcherrimin biosynthetic mutant Δ*yvmC-cypX* (YC800, 3) grown on biofilm-inducing media LBGM supplemented with 0.2 mM FeCl_3_. Plates were incubated at 30 °C and images were taken every 24 h over the course of 4 days. Scale bar, 10 mm. **b** Induction of the pulcherrimin biosynthesis operon during biofilm formation using the reporter strain bearing the transcriptional fusion P_*yvmC*_*-gfp* (LA20). Pellicle biofilms developed in LBGM and incubated statically at 30 °C. Pellicles were then harvested and cells examined under fluorescence microscopy every 24 hours over the course of 4 days. Representative phase and fluorescence images from each time point were shown. Scale bar, 10 μm. **c** Violin plots demonstrating the distribution of fluorescence expressed from wild-type cells bearing the P_*yvmC*_*-gfp* reporter (LA20). Cells were collected at 4 different time points during pellicle biofilm development (from 24 h to 96 h, as shown in **b** above). Each dot represents a single cell. Fluorescence pixel quantification of cells was carried out by using the MicrobeJ. Median values are represented by dashed horizontal lines. Solid lines represent standard deviation. **d** Activities of the *B. subtilis* strain bearing the transcriptional reporter P_*yvmC*_-*lacZ* (LA11). Pellicle biofilms of the reporter strain similarly developed and were collected, and β-galactosidase assays were performed. Average activities are representative of three biological replicates. Error bars represent standard deviation. M.U. Miller units.

To study the red-colored, iron-binding pigment pulcherrimin, we first constructed a mutant strain in *B. subtilis* (Δ*yvmC-cypX*, YC800) that abolished the production of this iron chelator, as well as a complementation strain (LA33, *yvmC-cypX* under the control of an IPTG-inducible promoter was integrated at the *amyE* locus in YC800). We spotted cells of the pulcherrimin mutant, the wild-type strain NCIB3610 (abbreviated as wild type hereafter), and the complementation strain on the iron-supplemented biofilm-inducing media LBGM + 0.2 mM FeCl_3_^45^. After incubation for 48 hours, a reddish halo surrounding the colony biofilms was clearly observed in both the wild-type and the complementation strains, but completely absent in the mutant (Fig. 1a). This suggests that the main component of the reddish pigment is pulcherrimin. Overproduction or loss of pulcherrimin does not seem to impact growth of *B. subtilis* cells since no difference in growth was observed among the wild-type, the pulcherrimin mutant, and the complementation strains (Supplementary Fig. 1).

Pulcherrimin production seems to increase over time during biofilm development (Fig. 1a). To learn about when and how the pulcherrimin biosynthetic operon (*yvmC-cypX*) is activated during biofilm development, we constructed two transcriptional reporters by fusing the promoter of the *yvmC* gene to the green fluorescent protein gene (*gfp*)(LA20), and to the β-galactosidase gene *lacZ* (LA11), and introduced the two reporter fusions into the wild-type strain, respectively. Biofilm pellicles of the reporter strain bearing P_*yvmC*_-*gfp* developed on LBGM, and were harvested every day over the course of 4 days and imaged using fluorescence microscopy. After measuring the mean fluorescence levels of hundreds of cells from each time point using MicrobeJ, we obtained the violin plots shown in Fig. 1c. The results show that there was a modest promoter activation in cells from the 24 h pellicles, while the peak activation was observed at 48 h. Cells from the 72 h and 96 h pellicles also displayed moderated fluorescence compared with the peak activation at 48 h. In addition, population heterogeneity was observed for the fluorescent reporter activation, with a subpopulation of cells displaying strong fluorescence while others displayed modest fluorescence (Fig. 1b, c). A similar expression profile, including peak activation, was observed when the P_*yvmC*_-*lacZ* reporter strain (LA11) was used and β-galactosidase assays conducted for pellicle samples similarly collected (Fig. 1d).

### Synthesis of pulcherriminic acid remains constitutive upon changing iron levels

**Fig. 2:**
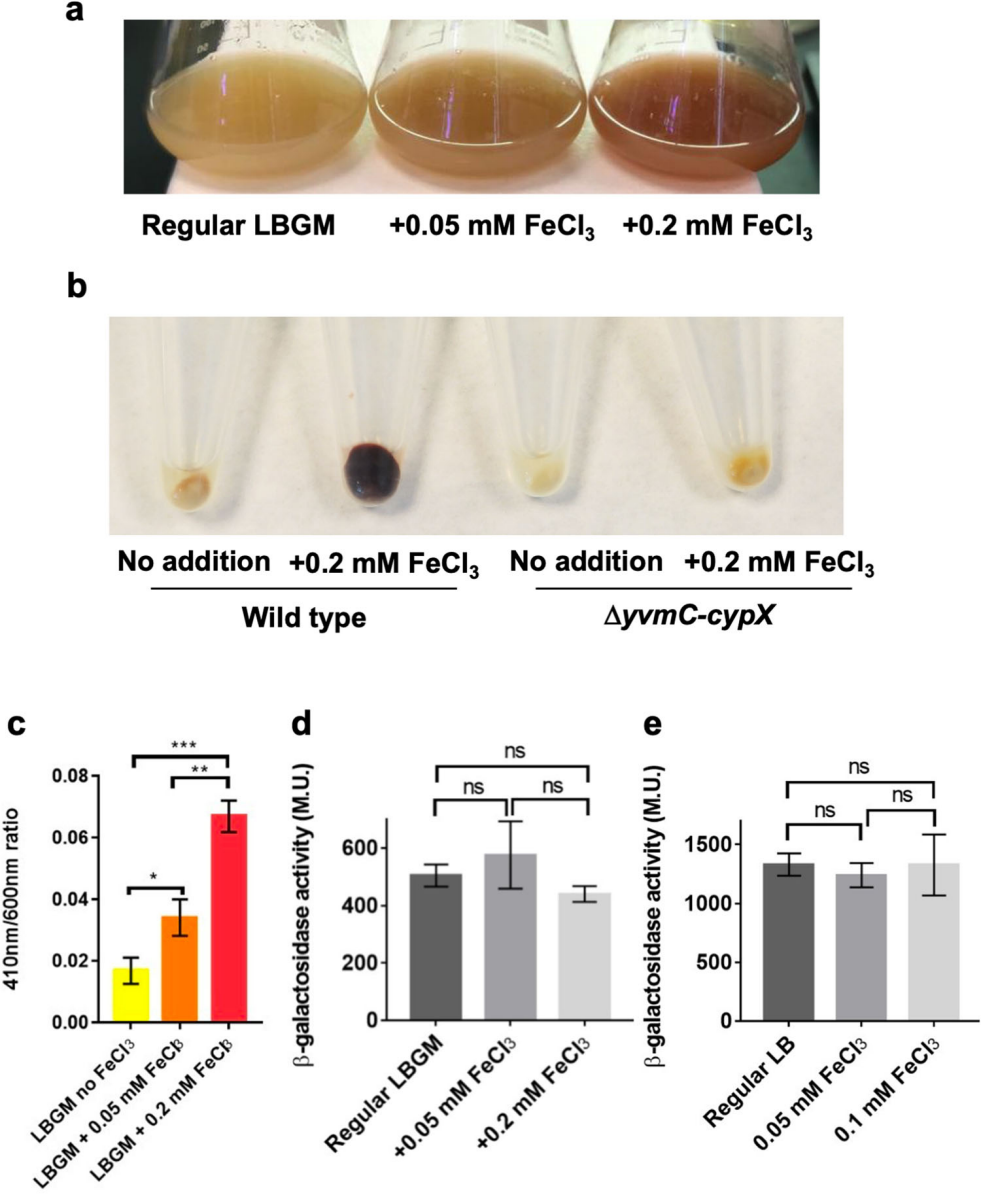
Addition of iron to growth media increases the yield of pulcherrimin, but not the activity of its biosynthetic genes. **a** Comparison of *B. subtilis* wild-type cell suspensions grown overnight under shaking in regular LBGM broth and in LBGM supplemented with 0.05 or 0.2 mM FeCl_3_. As the concentration of FeCl_3_ increases, so does the amount of reddish pigment. **b** Pellets obtained after harvesting cell suspensions of the wild type (left) and the pulcherrimin mutant (right) grown overnight in LBGM broth without or with the addition of 0.2 mM FeCl_3_. Pellets contained a mix of cells and pulcherrimin, which co-precipitate after centrifugation. **c** Results of indirect pulcherrimin quantification from cell pellets obtained after centrifugation of wild-type overnight cell suspensions from **a**. There is a significant increase in pulcherrimin production in samples supplemented with FeCl_3_ to a final concentration of 0.05 mM compared with the ones without FeCl_3_ supplementation (*p* = 0.0151, *); significantly higher pulcherrimin production was also observed when comparing samples supplemented with 0.2 mM FeCl_3_ to the ones without FeCl_3_ supplementation (*p* = 0.0002, ***). Averages are representative of three biological replicates. Error bars represent standard deviation. **d**, **e** Results of β-galactosidase assays for the pulcherrimin reporter P_*yvmC*_*-lacZ* under shaking (24 h, **d**) and pellicle (48 h, **e**) conditions in LBGM broth without or with FeCl_3_ supplementation at 0.05 or 0.1 mM. Averages are representative of three biological replicates. Error bars represent standard deviation. ns: statistically not significant. M.U. Miller units.

Even though pulcherrimin is an iron-binding molecule, the pulcherrimin biosynthetic operon, *yvmC-cypX*, is not known to be regulated by Fur, a master regulator for iron homeostasis and biosynthesis of other iron-binding molecules such as bacillibactin in *B. subtilis*^16^. Interestingly, however, when we grew wild-type cells in LBGM, and LBGM supplemented with 0.05 mM or 0.2 mM FeCl_3_, we observed higher pigment yield when higher amounts of FeCl_3_ were added to the media (Fig. 2a). FeCl_3_ supplementation alone did not lead to a significant difference in the color of the media (Supplementary Fig. 2). We harvested an equal number of cells from the above cultures and cell pellets showed strong color differences (note that pulcherrimin is largely insoluble and can be spun down by centrifugation) (wild type, Fig. 2b)^46–48^. Very little color difference was observed when the pulcherrimin mutant was used (Δ*yvmC-cypX*, Fig. 2b). This indicates that the color difference was due to different amounts of pulcherrimin present. We also measured the amount of insoluble pulcherrimin that co-precipitated with the cell pellets by converting it to soluble yellow-colored pulcherriminic acid. This was done by adding 2 M sodium hydroxide (NaOH) solution to the cell pellet containing pulcherrimin, followed by resuspension and measurements by spectrophotometer at 410 nm, which corresponds to the peak absorbance wavelength of pulcherriminic acid^24,49^. The results show that there is a direct correlation between increasing concentrations of FeCl_3_ in the media and higher pulcherrimin production (Fig. 2c). The amounts of free pulcherriminic acid in the supernatants of the same LBGM cultures supplemented with different amounts of FeCl_3_ were also measured. It was observed that the more FeCl_3_ was added to the media, the less free pulcherriminic acid was present in the supernatant (Supplementary Fig. 3). Collectively, the results above suggest that the pulcherrimin yield is significantly influenced by the iron levels in the external environment, even though its biosynthetic genes are not known to be regulated by the iron-responsive regulator Fur.

We then tested if iron concentrations in the media regulate the activity of pulcherrimin biosynthetic genes. We cultured the P_*yvmC*_-*lacZ* reporter strain under biofilm conditions (48 h development) as well as in shaking (24 h growth), in LBGM and LBGM supplemented with different amounts of FeCl_3_. The results show that the activities of the pulcherrimin biosynthetic genes did not respond to varying concentrations of ferric iron in the media in both biofilm pellicles and shaking cultures (Fig. 2d, e). Thus, iron concentrations in the media influence pulcherrimin yield without regulating the expression of its biosynthetic genes (e.g., regulating secretion of pulcherriminic acids). Another possibility, which we favor, is that the colorless pulcherriminic acid molecules (iron-less) are produced and secreted in large quantities irrelevant to the extracellular iron concentration but are increasingly converted to pulcherrimin (iron-bound) in the media in the presence of increasing amounts of ferric iron (Fig. 2c and Supplementary Fig. 3). To test this hypothesis, the spent supernatant from 3-day old wild-type pellicles was collected and filter sterilized. This cell-free supernatant, which contained free pulcherriminic acids, was split into 3 different tubes, each with addition of different amounts of FeCl_3_ (no addition, 0.05 mM and 0.2 mM). The results display a significant change in the color of the supernatants from light yellow to reddish-brown as the iron concentration increases (Supplementary Fig. 4). The color change corresponds to production of the pigment pulcherrimin. Therefore, this result seems to support our hypothesis above.

### Global transcription profiling suggests that pulcherrimin regulates genes involved in iron homeostasis and DNA damage response

**Fig. 3:**
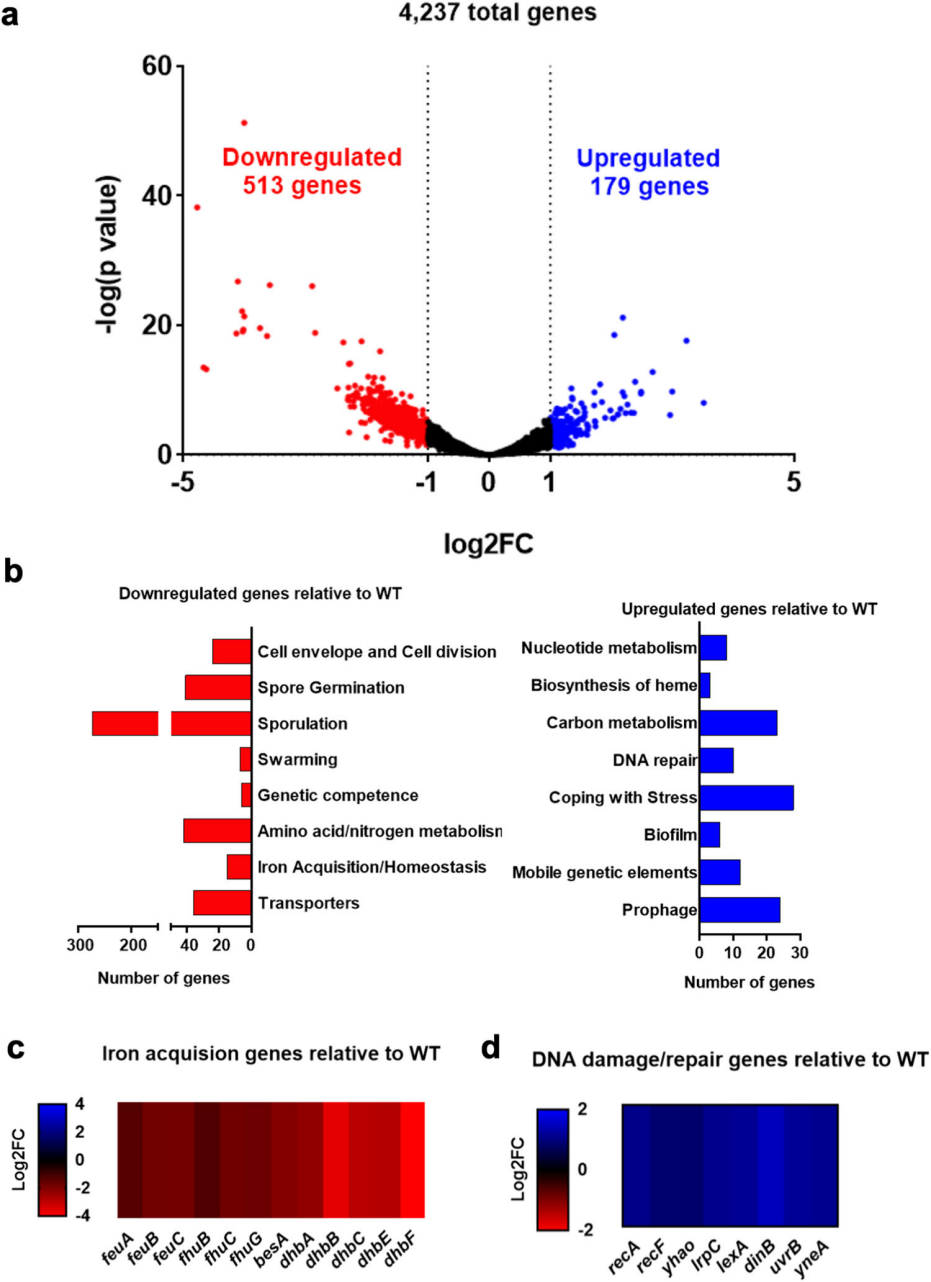
Global transcriptome analyses reveal differentially expressed genes in the pulcherrimin mutant relative to wild type. **a** Volcano plot depicting differentially expressed genes (blue and red dots) based on RNA-Seq and global transcriptome analyses. Downregulated genes with a log2FC of -1 or lower are depicted in red, and upregulated genes with a log2FC of 1 or higher are depicted in blue. Black dots represent genes not differentially expressed. **b** Significantly upregulated and downregulated genes were categorized according to known or predicted function. The graph shows the number of genes that fall into each category. Blue bars correspond to upregulated genes and red bars, downregulated genes. **c**, **d** Heatmaps displaying significantly downregulated genes for iron acquisition/homeostasis (**c**) and upregulated genes related to the DNA damage response (**d**) in the pulcherrimin mutant relative to wild type. RNA-seq transcriptome analysis was performed in samples from pellicle biofilms (collected at 72 h). Values are the average of three biological replicates per strain.

To have a better understanding of transcriptional regulation, we characterized the global transcriptome of the pulcherrimin mutant compared with the wild type under biofilm conditions. Pellicle biofilms of the wild type and the mutant were grown in LBGM. Pellicles were collected after 72 hours. Global transcription profiling was performed using RNA-Seq. Principal Component Analysis (PCA) suggested that in general, the wild-type replicates clustered separately from the pulcherrimin-mutant replicates (Supplementary Fig. 5). We applied a cutoff of log2 fold change (log2FC) of ±1 for significantly up- and downregulated genes, respectively. A volcano plot was generated where we could observe a total of 4237 genes retrieved from the RNA-Seq analysis, 513 of which significantly downregulated (Supplementary Table 1), and 179 upregulated (Supplementary Table 2) in the pulcherrimin mutant (Fig. 3a). Both sets of genes were categorized according to known or predicted function, and the results were summarized in Fig. 3b. In the pulcherrimin mutant, we could observe a much higher number of upregulated genes related to general stress response (“Coping with Stress”, 28 genes), as well as DNA damage response (“DNA repair”, 10 genes). On the other hand, a significant number of genes related to iron acquisition/homeostasis (15 genes) were downregulated in the pulcherrimin mutant. More strikingly, hundreds of sporulation genes were downregulated in the pulcherrimin mutant, implying that pulcherrimin has an influence on sporulation during biofilm development in *B. subtilis*. Lastly, we also performed the bioinformatic analysis on the raw RNA-seq data using the reference sequence for the pBS32 plasmid from NCIB3610^50^. Among the total 96 genes included in the analysis, only 4 genes were significantly downregulated and 5 upregulated (Supplementary Table 3 and Supplementary Fig. 6). We did not further analyze those differentially expressed genes on the pBS32 plasmid.

Pulcherrimin is known to be an iron chelator. Previous studies have shown that secreted pulcherrimin caused localized iron depletion in the media^21,51^. Results from our transcriptome profiling show that many iron homeostasis genes were downregulated in the pulcherrimin mutant, including the entire bacillibactin biosynthetic operon (*dhbABCEF*), bacillibactin transport genes (*feuABC*, *fhuBCG*) and the bacillibactin esterase gene *besA* (Fig. 3c). Bacillibactin is the main iron-scavenging molecule in *B. subtilis*, and it possesses extremely high affinity to iron ions^20,52^. BesA is a bacillibactin esterase, which catalyzes the hydrolysis of iron from bacillibactin and its release intracellularly^53^. Lastly, the operons *feuABC* and *fhuBCG* are responsible for uptake of bacillibactin bound to iron from the extracellular environment^54^. A plausible explanation for the above results is that in the wild-type biofilm (day 3 pellicle), cells experience iron limitation stress due to chelation of ferric iron by pulcherrimin in the media, thus increasing the expression of those iron-scavenging pathway genes. Indeed, localized iron depletion caused by pulcherrimin production has already been reported in the previously published study^21^. This result also supports the idea that pulcherrimin is not likely a siderophore used for iron acquisition by *B. subtilis*^21^.

Among the upregulated genes in the pulcherrimin mutant, we observed a significant number of DDR genes. Many of these genes are controlled by the DDR regulator LexA (*recA*, *yhaO*, *lexA*, *dinB*, *yneA,* and *uvrB*, Fig. 3d), and are part of the DDR pathway that is upregulated when cells experience DNA damage^41^. We previously showed that in the wild-type *B. subtilis* biofilm, DDR genes were modestly activated due to accumulation of reactive oxygen species (ROS) derived from metabolism^35^. This result indicates that in the absence of pulcherrimin production, more significantly elevated DNA damage may occur in cells in the mature biofilm.

### Pulcherrimin protects cells from DNA damage

**Fig. 4:**
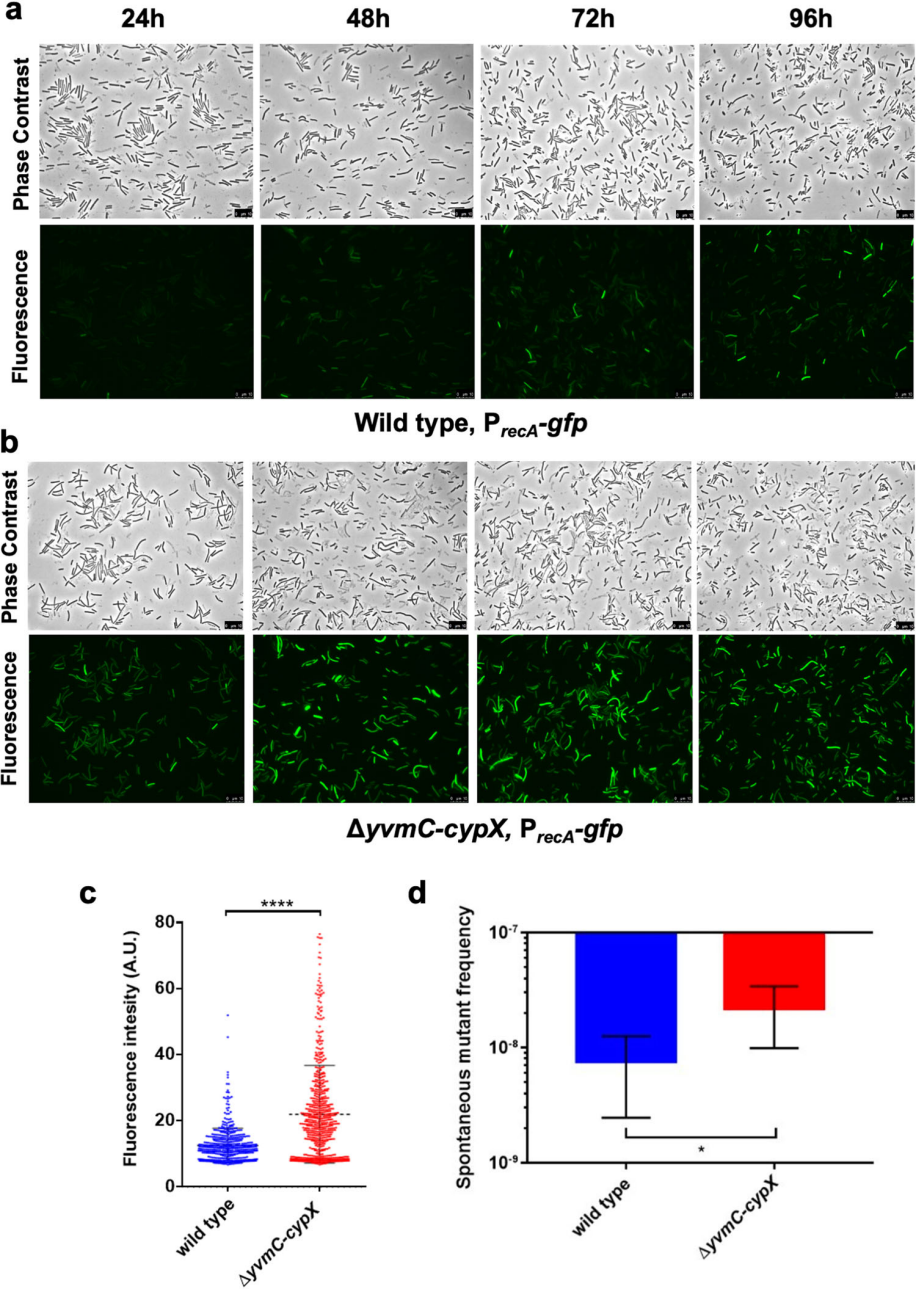
The DNA damage response is elevated in the pulcherrimin mutant. **a**, **b** Phase contrast and fluorescence microscopy images of the wild type (LA174, top panels, **a**) and pulcherrimin mutant (LA224, bottom panels, **b**) bearing the P_*recA*_*-gfp* reporter for DNA damage response were taken every 24 h during pellicle biofilm development. Scale bar, 10 μm. **c** Violin plots portraying the distribution of wild-type and pulcherrimin-mutant cells according to their quantified fluorescence collected at 48 h of pellicle biofilm development in (**a**, **b**). Each dot represents a single cell. Fluorescence pixel quantification of cells was carried out by using the MicrobeJ plugin of Image J. Median values are represented by dashed horizontal lines. Solid lines represent standard deviation. There is a significant difference in the fluorescence levels between the wild type and the pulcherrimin mutant (*p* < 0.0001, ****). **d** The pulcherrimin mutant generates more spontaneous rifampicin mutants compared with the wild type (*p* = 0.02, *). 24 h cultures in LBGM supplemented with 0.2 mM FeCl_3_ of the wild-type strain and the pulcherrimin mutant were plated separately onto rifampicin agar plates (5 μg/mL) and incubated overnight. Experiments were performed in biological triplicate and error bars represent standard deviation.

Our RNA-Seq results indicated that multiple DDR genes were upregulated in the pulcherrimin mutant. To further investigate this, we constructed a DDR reporter by fusing the promoter of *recA*, a well-known DDR gene, with *gfp*. The reporter fusion was introduced, by integration at the *amyE* locus, into the wild type (LA174) and the pulcherrimin mutant (LA224), respectively. Pellicle biofilms developed in LBGM, and were collected every day over the course of 4 days and imaged using a fluorescence microscope. The wild-type reporter strain displayed increasing fluorescence over time (Fig. 4a), which was expected due to increasing accumulation of toxic molecules derived from metabolism that could damage DNA as the biofilm ages^35^. Interestingly, the pulcherrimin mutant displayed much higher fluorescence compared with the wild type at each of the four time points that the samples were collected (Fig. 4b). We quantified the fluorescence of hundreds of individual cells from both the wild type and the pulcherrimin mutant collected at 48 h using MicrobeJ^55^. Figure 4c is the violin plot generated from the quantifications, where it shows that the values of fluorescence representing the activities of the DDR reporter *P*_*recA*_*-gfp* are significantly higher in the pulcherrimin mutant (red) than in the wild type (blue). These results indicate that in the mature biofilm, cells of the pulcherrimin mutant likely undergo elevated stress that leads to more DNA damage compared with the wild type.

**Fig. 5:**
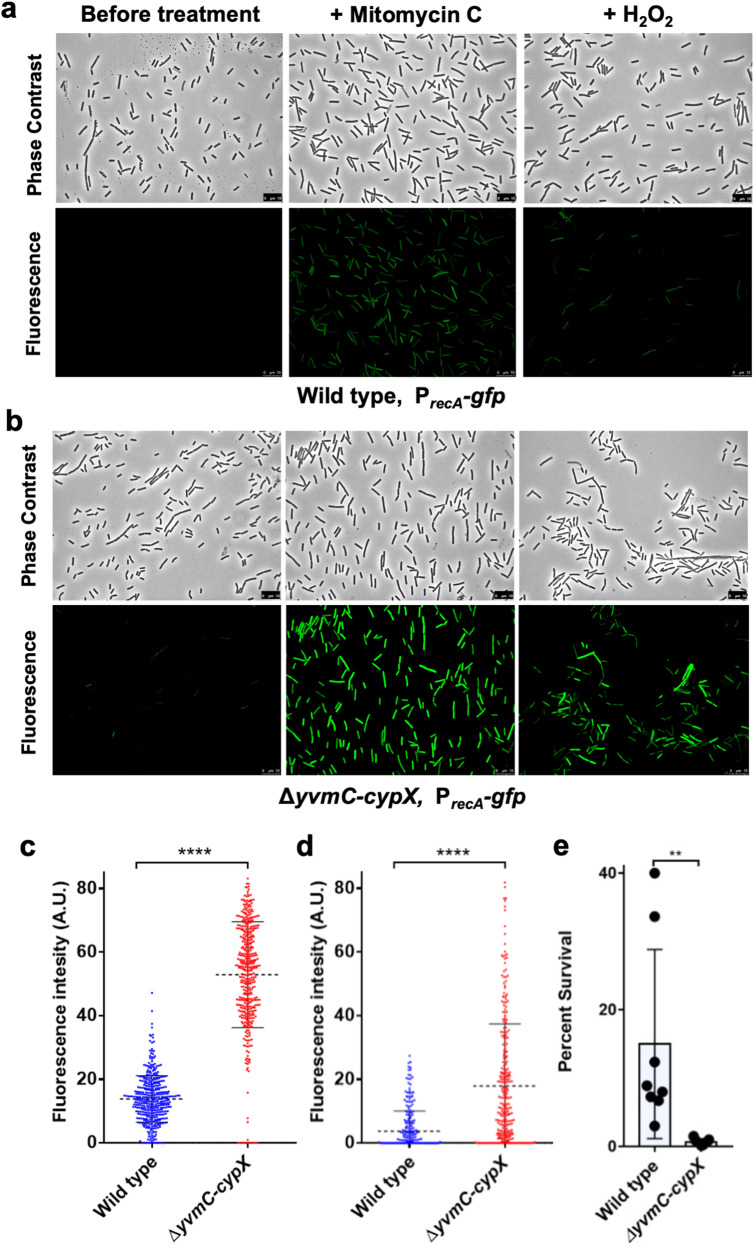
The pulcherrimin mutant is more sensitive to DNA-damaging agents compared with the wild type. **a**, **b** Phase contrast and fluorescence microscopy images obtained from cultures of the wild type (top panels, **a**) and the pulcherrimin mutant (bottom panels, **b**) bearing the P_*recA*_*-gfp* reporter before and after challenge with the DNA-damaging agents mitomycin C (0.25 μg/mL) or H_2_O_2_ (100 μm) for one hour. Scale bar, 10 μm. **c**, **d** Single-cell fluorescence was quantified for images in A (treated with mitomycin C, panel C) and in B (treated with H_2_O_2_, **d**) and a violin plot was generated. Each dot in the graph represents a single cell. Median values are represented by dashed horizontal lines. Solid lines represent standard deviation. The pulcherrimin mutant (red) displayed significantly higher fluorescence than the wild type (blue) in both treatments (*p* < 0.0001, ****). **e** Percent survival of wild-type cells after treatment with H_2_O_2_ (final concentration at 10 mM) for 10 minutes under shaking conditions is significantly higher when compared with the pulcherrimin mutant under the same treatment (*p* = 0.0039, **). Average is representative of three independent experiments with a total of 8 biological replicates. Each dot represents an individual value for each replicate. Error bars represent standard deviation.

To further assess the implication of the above results, the wild-type, and the pulcherrimin-mutant reporter strains were challenged with two different DNA-damaging agents. One of them was mitomycin C, an antibiotic known to cause DNA double-stranded breaks and widely used to study the DDR in *B. subtilis*, and the other was hydrogen peroxide (H_2_O_2_), a well-known ROS that causes damage to several cellular structures including DNA^56,57^. The assay consisted of growing cells under shaking conditions in LB broth for 2 hours followed by mitomycin C or H_2_O_2_ challenge for one hour at the final concentration of 0.25 μg/mL and 100 μm, respectively. Controls corresponded to untreated samples. After treatment, cells were collected, washed twice with PBS, and imaged under a fluorescence microscope. The P_*recA*_-*gfp* reporter in the pulcherrimin mutant was found to display substantially higher fluorescence upon both DNA-damaging treatments compared with the wild type (Fig. 5a–d). These results suggest that lack of pulcherrimin rendered the mutant much more sensitive to external DNA-damaging agents than the wild type. We thus conclude that pulcherrimin plays a role in protecting cells by lowering DNA damage caused by both internal and external stresses.

To test whether levels of DNA damage are physically elevated in the pulcherrimin mutant, we applied assays of spontaneous rifampicin resistance frequency as a measurement for DNA mutation rate and levels of DNA damage^58^. To do so, we grew both the wild type and the pulcherrimin mutant for 24 h in LBGM supplemented with 0.2 mM FeCl_3_. We chose to culture the cells in an iron-overloaded medium so they would be more prone to oxidative stress through the Fenton reaction and consequently more DNA damages if a protective mechanism is lacking. Cells of each strain were then plated onto LB agar supplemented with rifampicin (5 μg/mL) and incubated overnight. Spontaneous rifampicin-resistant mutants appeared on the plates of the wild-type strain as expected. Interestingly, on the plates of the pulcherrimin mutant, the number of spontaneous rifampicin-resistant colonies appeared at a much higher rate than the wild type (Fig. 4d). This result supports our hypothesis that in the wild type, pulcherrimin protects cells by physically lowering DNA damage.

To further test if there are differences in cell survival under severe DNA damage stress between the wild type and the pulcherrimin mutant, we grew cells overnight in LBGM supplemented with 0.2 mM FeCl_3_ (to ensure high pulcherrimin yield in the wild type; Fig. 2a). Each overnight culture was then split into two flasks. In one of the flasks, H_2_O_2_ was added at a final concentration of 10 mM for 10 min with shaking. The second flask, as a control, was not exposed to H_2_O_2_. Untreated and H_2_O_2_-treated cultures were both plated on LB agar plates and incubated overnight at 37 °C. CFUs were counted the next day and the percent survival calculated for both strains. The average percent survival of the pulcherrimin mutant after exposure to H_2_O_2_ was approximately 20 times lower compared with the wild type (Fig. 5e). This result supports the idea that pulcherrimin protects cells and allows better survival of the cells under severe DNA damage stress.

### Pulcherrimin production and DNA damage appear to be anticorrelated in cells in the biofilm

**Fig. 6:**
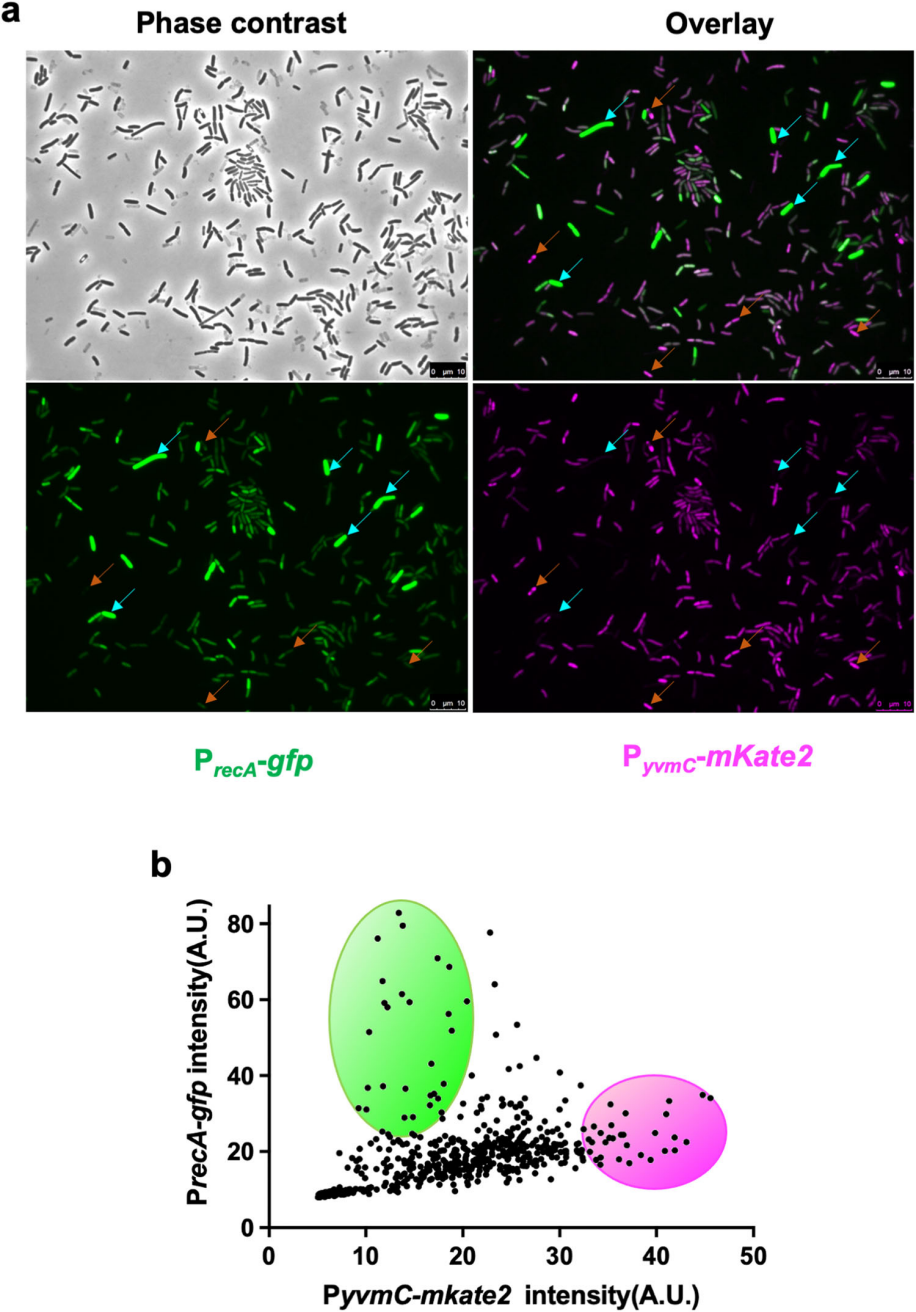
Strong pulcherrimin producers and cells with significantly elevated DNA damage response in the biofilm appear to anticorrelate. **a** Wild-type *B. subtilis* carrying the dual reporter P_*recA*_*-gfp* and P_*yvmC*_*-mkate2* (LA233) was cultured in LBGM for pellicle biofilm development and fluorescence imaging was performed on samples collected after 48 h of development. A representative image for each fluorescence channel is displayed, as well as an overlay image of both GFP and mKate2. Arrows in cyan represent cells with high GFP and low mKate2 expression, while arrows in orange represent those with high mKate2 and low GFP expression. Scale bar, 10 μm. **b** Dot plot representing the distribution of cells according to their fluorescence levels for both reporters. Each dot represents a single cell. The green circle highlights the subpopulation of cells that display high fluorescence for the P_*recA*_*-gfp* reporter and low fluorescence for the P_*yvmC*_*-mkate2* reporter. The magenta circle highlights the subpopulation with high P_*yvmC*_*-mkate2* and low P_*recA*_*-gfp* activities.

We constructed a dual reporter strain (LA233) carrying both the DDR reporter P_*recA*_*-gfp* as a proxy for DNA damage, and the P_*yvmC*_*-mKate2* reporter for pulcherrimin production. We cultured this dual reporter strain in LBGM for pellicle development, harvested the pellicles after 48 hours (mature biofilm), mildly sonicated the pellicles to disrupt the bundles and chains, and imaged the cells under the fluorescence microscope applying the corresponding wavelengths for each reporter (Fig. 6a). We then quantified the fluorescence for each reporter in individual cells and plotted the results in a dot plot graph where the DDR reporter’s green fluorescence is displayed in the y axis, and the pulcherrimin reporter is on the *x* axis in fluorescent magenta (Fig. 6b). Each dot in the panel corresponds to a single cell bearing both reporters. The results depicted here show a significant amount of overlap among cells that displayed moderate fluorescence for both reporters, suggesting that a large proportion of cells in a mature biofilm express both reporters at intermediate levels. However, it is important to note that cells displaying high green fluorescence for the DDR reporter had very low magenta fluorescence for the pulcherrimin reporter (cells pointed by cyan-colored arrows in panels in Fig. 6a), and vice-versa (cells indicated by orange-colored arrows in the overlay image in Fig. 6a). Thus, we observed that high pulcherrimin production anti-correlates with levels of DDR and probably of physical DNA damage. This, again, supports our hypothesis that pulcherrimin can protect cells from DNA damage.

### Pulcherrimin lowers ROS accumulation

Our results suggest that pulcherrimin provides protection against DNA damage (Figs. 3, 4). We wanted to further elucidate what could be causing this effect. ROS such as H_2_O_2_, superoxide radicals, and hydroxyl radicals, are byproducts of cell metabolism and are known to cause DNA damage such as double-stranded breaks^30,59,60^. We previously showed that ROS increasingly accumulates during biofilm development and triggers DDR in cells in the *B. subtilis* biofilm^35^. We thus hypothesized that pulcherrimin may protect cells against ROS and thus ROS-induced DNA damage. We decided to directly measure ROS levels in the wild type and the pulcherrimin mutant by utilizing a kit for total ROS measurements (Enzo Biosciences), which includes a cell-permeable and non-fluorescent dye that, once in contact with ROS (hydrogen peroxide or hydroxyl radicals), generates green fluorescence. Cells can then be imaged using a fluorescence microscope with a standard green filter (490/525 nm).

**Fig. 7:**
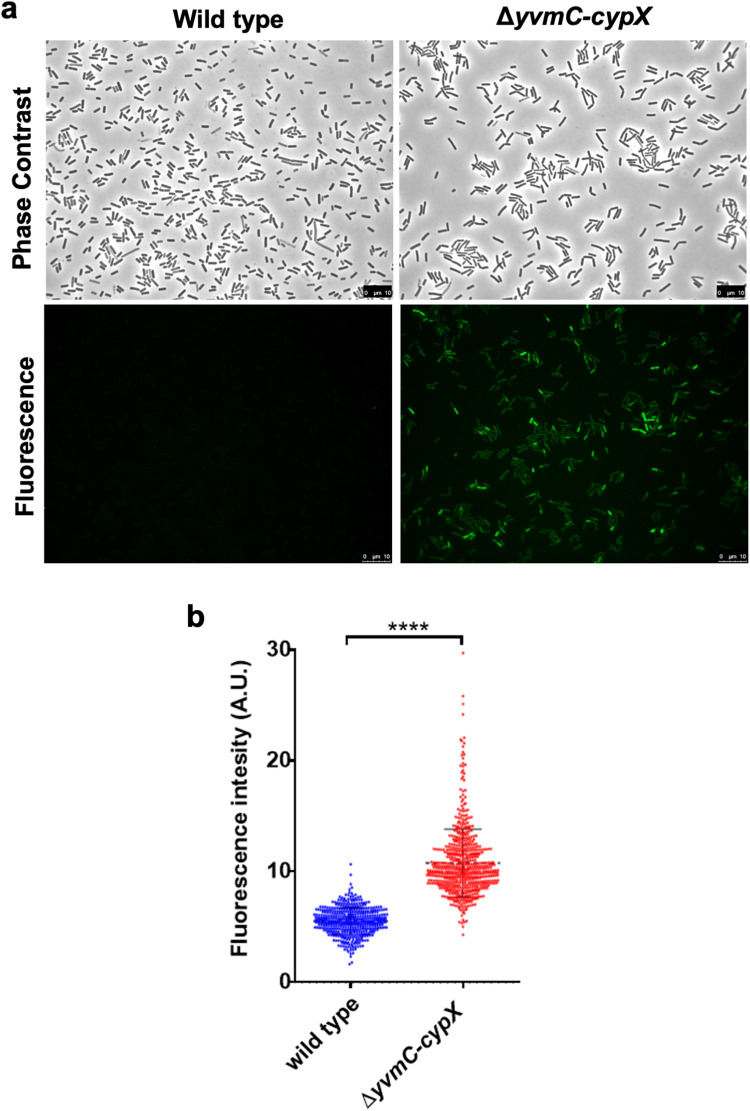
Pulcherrimin production reduces ROS accumulation. **a** Accumulation of different ROS species (H_2_O_2_, peroxynitrite, and/or hydroxyl radicals) in the pulcherrimin mutant and the wild type was visualized by using a ROS detection kit. Overnight cultures of the pulcherrimin mutant and the wild type growing in LBGM supplemented with 0.2 mM FeCl_3_ were harvested, cells were stained with the ROS dye, and after incubation, images were taken under a fluorescence microscope. Scale bar, 10 μm. **b** Fluorescence quantification of the cells in the images (in **a**) was performed using the MicrobeJ plugin of Image J. Each dot in the violin plot corresponds to a single cell. The median values are represented by dashed horizontal lines. A statistically significant difference was observed between the wild type and the pulcherrimin mutant (*p* < 0.0001, ****).

Cells of the wild type and the pulcherrimin mutant were grown overnight in LBGM supplemented with 0.2 mM FeCl_3_ to enhance pulcherrimin yield. Cells were then incubated with the dye for 30 minutes in the dark, followed by three washes with PBS to remove excess dye, and then imaged. Figure 7a corresponds to representative fluorescence microscopy images acquired for each sample, where the green fluorescence levels in the pulcherrimin mutant were clearly higher than in the wild type. Fluorescence of individual cells was also quantified using Image J and plotted in a violin plot, which exhibited a significant difference between the two strains (Fig. 7b). These results suggest that pulcherrimin is able to lower ROS accumulation and thus prevent damaging levels of oxidative stress to happen in the cells.

### Pulcherrimin production could reduce iron toxicity by lowering iron levels

The results above provide both direct and indirect evidence that pulcherrimin reduces the oxidative stress in the cells and protects them from DNA damage. Finally, we can speculate that the reason why pulcherrimin can provide protection is probably due to its ability to sequester iron and lower iron bioavailability both outside the environment and inside the cells. Robust biofilm formation by *B. subtilis* demands excessive amounts of iron in the media for reasons that are still not well understood^33,61^. Iron overload is known to cause toxicity because it triggers the Fenton reaction^62^. Thus, we expect that iron sequestration by pulcherrimin leads to reduced Fenton reactions, less accumulation of hydroxyl radicals, and consequently, less DNA damage. Our RNA-Seq results show that lack of pulcherrimin led to downregulation of iron homeostasis genes, suggesting that pulcherrimin produced by the wild type not only chelates extracellular iron, but also indirectly lowers intracellular iron levels.

**Fig. 8:**
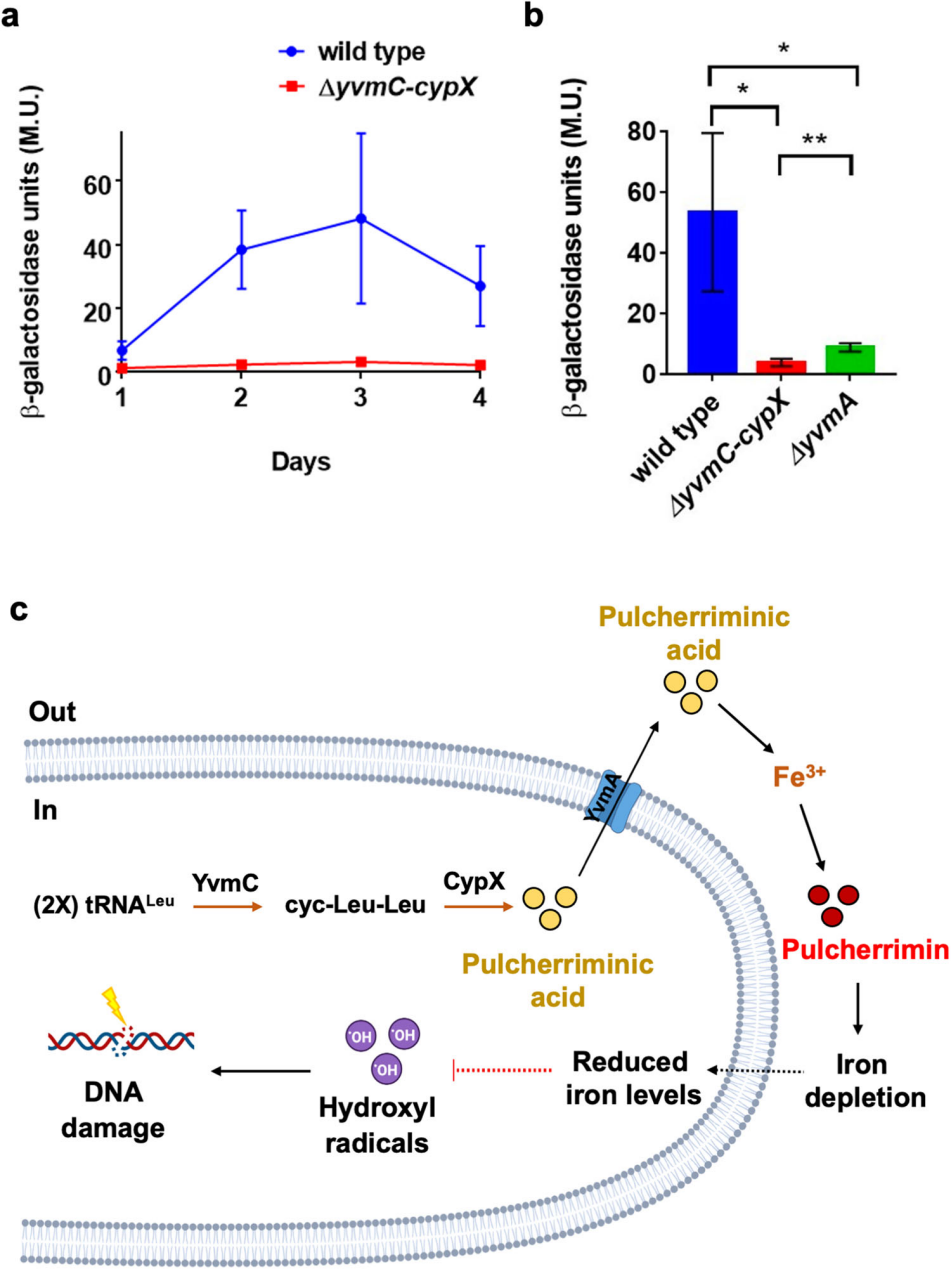
Pulcherrimin production could reduce iron toxicity by lowering iron levels. **a** The promoter activity for the bacillibactin biosynthetic operon was compared between the pulcherrimin mutant (LA148) and the wild type (YQ141) by using the transcriptional reporter P_*dhbA*_*-lacZ*. β-galactosidase assays were performed using pellicle biofilms of the two reporter strains collected every 24 hours over the course of 4 days. **b** A mutant for the pulcherrimin transporter (Δ*yvmA*) was also tested for the activation of the P_*dhbA*_*-lacZ* reporter (LA262). Pellicle biofilms of the three reporter strains were harvested after 72 h, and β-galactosidase assays were performed accordingly. Results show a significant decrease in promoter activation in Δ*yvmA* compared with the wild type (*p* = 0.0417, *), but higher activation compared with the pulcherrimin mutant (*p* = 0.0097, **). **c** A working model for the function of pulcherrimin during *B. subtilis* biofilm development as an iron-buffering molecule. This model shows that pulcherriminic acid molecules are produced intracellularly via two enzymatic reactions carried out by YvmC and CypX, respectively, using tRNA-charged leucine as the substrate. Pulcherriminic acid molecules are exported, via a dedicated transporter YvmA, to the extracellular environment where it binds to free ferric iron ions and turns into the reddish pigment pulcherrimin. Because iron-bound pulcherrimin is insoluble, iron depletion occurs extracellularly upon large quantities of secreted pulcherriminic acid molecules competitively chelating iron. This depletion likely results in lowered intracellular iron levels, which then leads to reduced Fenton reactions and less accumulation of DNA-damaging hydroxyl radicals. Thus, pulcherrimin production acts as an extracellular iron-buffering mechanism to reduce ROS production, consequently lowering oxidative stress and preventing DNA damage in *B. subtilis*. Model created with BioRender.com.

To test the above hypothesis that by sequestering extracellular iron, pulcherrimin production can lower intracellular levels of iron, a transcriptional reporter with the promoter for the bacillibactin operon, whose expression highly anti-correlates with intracellular iron levels, fused to *lacZ* was constructed (P_*dhbA*_-*lacZ*), and introduced into the wild type (YQ141) and the pulcherrimin mutant (LA148), respectively. Pellicle biofilms of the two reporter strains developed and were then harvested daily over the course of 4 days. β-Galactosidase assays were performed on collected samples and results are shown in Fig. 8a. It is evident that the pulcherrimin mutant displayed much lower activities of the P_*dhbA*_-*lacZ* reporter when compared with the wild type at every time point measured. This result supports the hypothesis that pulcherrimin production in the wild type significantly lowered intracellular iron levels and thus triggered upregulation of iron acquisition through bacillibactin and possibly other siderophores as well. It was reported that pulcherrimin production causes iron depletion in the surroundings so that *B. subtilis* needs to scavenge more iron from the extracellular environment^21^. Our findings are consistent with the above-published study and further suggest that this also leads to lowered intracellular iron levels.

To further confirm that the observed difference in the P_*dhbA*_-*lacZ* reporter activity between the wild type and the pulcherrimin mutant is caused by the formation of pulcherrimin extracellularly, and consequently iron depletion, we introduced the same reporter into the pulcherrimin transporter mutant, Δ*yvmA* (LA262). This *yvmA* gene mutation significantly reduces the secretion, but not biosynthesis, of pulcherriminic acid to the extracellular environment, as less pulcherrimin is observed in the media when compared with the wild type^21,26^. Pellicle biofilms were similarly collected, and β-galactosidase assays performed to compare the activities of the P_*dhbA*_-*lacZ* reporter in the wild type, the pulcherrimin biosynthetic mutant, and the pulcherrimin secretion mutant. The results show that both mutants (Δ*yvmC-cypX* and Δ*yvmA*) displayed a much lower promoter activation when compared with the wild type, with the pulcherrimin biosynthetic mutant (Δ*yvmC-cypX*) having the lowest promoter activation of all three (Fig. 8b). This confirms that the reason why the P_*dhbA*_-*lacZ* reporter displays different activities between the wild type and the pulcherrimin mutant is indeed because of the formation of pulcherrimin extracellularly.

Together, the results described above further confirm that pulcherrimin production leads to extracellular iron depletion in wild-type biofilms as previously shown^21^. The iron depletion likely also significantly lowers intracellular iron levels because the promoter for the bacillibactin operon is strongly upregulated in the wild type compared with the pulcherrimin mutant, indicating an increased need for iron acquisition when pulcherrimin is present. To this end, we have presented several lines of evidence to support our working model on how pulcherrimin can function as an antioxidant to protect cells from oxidative stress and DNA damage during *B. subtilis* biofilm development (Fig. 8c).

## Discussion

In this study, we aimed to investigate the role of the secreted iron chelator pulcherrimin during *B. subtilis* biofilm development. Based on our findings, we proposed a model for the function of pulcherrimin mainly as an antioxidant in *B. subtilis* (Fig. 8c). Pulcherriminic acid is produced inside the cell and is secreted to the extracellular environment by the dedicated transporter YvmA. If ferric iron ions are readily available, pulcherriminic acid molecules bind to them and turns into pulcherrimin. Because pulcherrimin is a largely insoluble molecule, it depletes the ferric iron ions when the precipitated complex accumulates in the extracellular environment. This iron depletion acts as a buffering mechanism that prevents too much iron from going into the cell. The cells are temporarily starving for iron when pulcherrimin is produced, which in this case is beneficial because the Fenton reaction is minimized and less hydroxyl radicals are generated intracellularly. Less hydroxyl radicals mean less DNA damage. Therefore, pulcherrimin, by lowering the ferric iron levels extracellularly, indirectly prevents cells from undergoing high levels of oxidative stress through the production of hydroxyl radicals.

Iron sequestration by pulcherrimin, the consequent localized iron depletion, and the reported growth arrest may initially sound counter intuitive. However, we want to point out that first, it is known that robust biofilm formation by *B. subtilis* demands hundreds of times more iron than what is needed for normal growth to be supplemented in the media^33^. Iron toxicity associated with biofilm formation is thus a predictable negative impact that cells are yet to encounter and overcome. Second, biosynthesis of pulcherrimin is tightly associated with biofilm development; pulcherrimin is produced to play a role in iron sequestration and protection against oxidative stress only in the stage of biofilm maturation, during which rapid growth of individual cells is no longer a priority, as compared to dealing with increasingly elevated oxidative stresses in the biofilm community. We speculate that one of the important functions of pulcherrimin is to alleviate oxidative stress in mature and aged biofilms where it directly chelates the ferric iron in the biofilm matrix and indirectly lowers intracellular iron levels to reduce the formation of hydroxyl radicals that could damage the DNA and other biomolecules. This could also explain why a large number of sporulation genes were downregulated in the pulcherrimin mutant as seen in our RNA-Seq results. *B. subtilis* cells have developed multiple checkpoint mechanisms to ensure DNA quality and remove any DNA damage before commencing sporulation^63–65^. In the pulcherrimin mutant, due to elevated DNA damages, cells may decide to stall sporulation until those damages are repaired. Alternatively, decreased sporulation gene expression we observed could simply be due to the reported growth arrest affect by pulcherrimin^21^.

While pulcherriminic acid (a dipeptide) is thought to be soluble, it has been implicated that, once bound to iron, pulcherrimin can form supramolecular assemblies among ferric iron atoms and precipitate^48^. These pulcherrimin assemblies become large agglomerates, making it non-diffusible and trapped within the biofilm matrix. It has been previously reported that the biofilm matrix deposits tens of times more iron when compared with that in the media^33,34^. However, in addition to the potential iron toxicity, it was also unclear to us how these iron ions are present in the matrix and in what form. Pulcherrimin would allow excess amounts of iron to be associated with the matrix in the form of supramolecular assemblies, and simultaneously prevent the negative impact of iron overload in the biofilm. Our unpublished evidence suggests that when the biofilms mature, large amounts of pulcherriminic acid have been produced and secreted to the environment. It depends on how much iron is available in the media that some proportions of secreted pulcherriminic acids are chemically converted to iron-bound pulcherrimin. It is thus likely that both the soluble iron-free pulcherriminic acids and insoluble iron-bound pulcherrimin coexist in the environment with a varying ratio that is dependent on iron availability in the media. Any potential new function of the iron-free pulcherriminic acid, independent from the iron-bound pulcherrimin has yet to be investigated, and remains extremely interesting to us. The last interesting note that we would like to pursue in the future is whether pulcherrimin can function as a “shared good” for cells in the biofilm community. We have seen initial evidence of heterogeneous activation of pulcherrimin biosynthetic genes in cells in the biofilm and possible molecular regulations responsible for this heterogeneity. It will be interesting to see if pulcherrimin could function in a way similar to exopolysaccharides and matrix proteins that are produced by a subpopulation of cells but are shared by the entire biofilm community^1^.

## Methods

### Bacterial strains and media

Table 1 lists all the strains, plasmids, and oligonucleotides utilized in this study. *Bacillus subtilis* NCIB3610, *B. subtilis* PY79, *Escherichia coli* DH5α, and derived strains were cultured in lysogeny broth (LB) (10 g/L tryptone, 5 g/L yeast extract, and 10 g/L NaCl) at 37 °C. Colony and pellicle biofilms were grown statically at 30 °C in LBGM (LB broth supplemented with final concentrations of 1% glycerol (v/v) and 100 μM MnSO_4_) broth or agar (1.5% w/v), respectively^45^. Restriction enzymes utilized in this study for molecular cloning were purchased from New England Biolabs (NEB, Ipswich, MA, USA). Reagents and chemicals were either purchased from Sigma-Aldrich (St. Louis, MO, USA) or Fisher Scientific (Agawam, MA, USA). Oligonucleotides were purchased either from Genewiz (South Plainfileld, NJ) or Eurofins Genomics (Louisville, KY, USA). Sanger DNA sequencing was also performed by either company. When needed, antibiotics were applied at the following concentrations: 100 μg/ml of spectinomycin, 1 μg/ml of erythromycin, 10 μg/ml of tetracycline, 50 μg/ml of kanamycin, and 10 μg/ml of chloramphenicol for *B. subtilis*. All *E. coli* DH5α strains were grown in LB broth supplemented with 100 μg/ml of ampicillin.

**Table 1:** Strains, plasmids, and oligonucleotides used in this study.

Strains	Description	Reference
3610	An undomesticated strain of *B. subtilis*, capable of forming biofilms	^5^
DH5α	*E. coli* strain for molecular cloning	Invitrogen
PY79	a competent laboratory strain of *B. subtilis* used for genetic manipulation	^67^
YC800	Δ*yvmC-cypX::tet*^*R*^ in 3610	this study
LA11	*amyE::P*_*yvmC*_*-lacZ, chl*^*R*^ in 3610	this study
LA20	*amyE::PyvmC-gfp, chl*^*R*^ in 3610	this study
LA33	Δ*yvmC-cypX::tet*^*R*^ *amyE::*P_*spank*_*-yvmC-cypX, spec*^*R*^ in 3610	this study
YQ141	*amyE::P*_*dhbA*_*-lacZ, spec*^*R*^ in 3610	^33^
LA148	Δ*yvmC-cypX::tet*^*R*^ *amyE::P*_*dhbA*_*-lacZ, spec*^*R*^ in 3610	this study
LA174	*amyE::P*_*recA*_*-gfp, spec*^*R*^ in 3610	this study
LA224	Δ*yvmC-cypX::tet*^*R*^ *amyE::P*_*recA*_*-gfp, spec*^*R*^ in 3610	this study
LA233	*amyE::P*_*recA*_*-gfp, spec*^*R*^ *lacA::P*_*yvmC*_*-mkate2, erm*^*R*^ in 3610	this study
LA208	Δ*yvmA::kan*^*R*^ in 168	BKK35090
LA247	Δ*yvmA::kan*^*R*^ in 3610	this study
LA262	Δ*yvmA::kan*^*R*^*. amyE::P*_*dhbA*_*-lacZ, spec*^*R*^ in 3610	this study
**Plasmids**		
LA2	pYC121, *amyE::P*_*yvmC*_*-gfp, amp*^*R*^ in DH5α	this study
LA9	pDG268, *amyE::P*_*yvmC*_*-lacZ, amp*^*R*^ in DH5α	this study
LA22	pDR111, *amyE::P*_*spank*_*-yvmC-cypX, amp*^*R*^ in DH5α	this study
LA72	pYC121, *amyE::P*_*yvmC*_*-mkate2, amp*^*R*^ in DH5α	this study
LA97	pDG268, *amyE::P*_*katE*_*-lacZ, amp*^*R*^ in DH5α	this study
LA163	pYC211, *amyE::P*_*recA*_*-gfp, amp*^*R*^ in DH5α	this study
LA186	pDR183, *lacA::P*_*yvmC*_*-mkate2, amp*^*R*^ in DH5α	this study
pDG268	*amyE* integration vector with a promoter-less *lacZ, spec*^*R*^*, amp*^*R*^	^68^
pDR111	*amyE* integration vector with an IPTG-inducible promoter*, spec*^*R*^*, amp*^*R*^	^69^
pDR183	*lacA* integration vector, *erm*^*R*^*, amp*^*R*^	^70^
pYC121	*amyE* integration vector with a promoter-less *gfp, chl*^*R*^*, amp*^*R*^	^71^
pYC211	*amyE* integration vector with a promoter-less *gfp, spec*^*R*^*, amp*^*R*^	this study
pYC251	*amyE* integration vector with an IPTG-inducible promoter *mkate2, chl*^*R*^*, amp*^*R*.^	^37^
pDG1730	*amyE* integration vector with amp^r^, spec^r^, and erm^r^	^72^
**Primers**		
P_*yvmC*_-F (for pYC121 fusion)	5’ GTACGAATTCGCGTGTAAAAAGTGTGTGGAAAATGTG 3’	
P_*yvmC*_-R (for pYC121 fusion)	5’ GTACAAGCTTCTCATTCACCCCTAAAACTTATCCCGC 3’	
P_*yvmC*_-F (for pDG268 fusion)	5’ GTACGAATTCGCGTGTAAAAAGTGTGTGGAAAATGTG 3’	
P_*yvmC*_-R (for pDG268 fusion)	5’ GTACGGATCCCTCATTCACCCCTAAAACTTATCCCGC 3’	
*yvmC*-F	5’ GTACGCTAGCAAGGAGGAACTACTATGACCGGAATGGTAACGG 3’	
*cypX*-R	5’ GTACGCATGCTTATGCCCCGTCAAACGCAACG 3’	
*mkate2*-F	5’ GTACGCATGCATGGATTCAATAGAAAAGGTAAGCG 3’	
*mkate2*-R	5’ ACTGGTCTGATCGGATCCTTAT 3’	
P_*recA*_-F	5’ GTACGAATTCTCGGCATTTCCGCAAATGG 3’	
P_*recA*_-R	5’ GTACGTCGACTCTATTTTTTCCTCCTTTATG 3’	
*yvmC*-P1	5’ AACAGTGTATAAGATCAATACTGCCTTTGATTTTGTATGA 3’	
*yvmC*-P2	5’ CAATTCGCCCTATAGTGAGTCGTCTCATTCACCCCTAAAACTT 3’	
*cypX*-P1	5’ CCAGCTTTTGTTCCCTTTAGTGAGTAGAATTCCAAAGGTCTCTCCC 3’	
*cypX*-P2	5’ TTTGTGCCAAAAACTTGCGGACCGCTCCGGGGGTCGGGTC 3’	

### Bacterial strain construction

The single insertional deletion mutant *yvmA::kan* (BKE40190) used in this study (listed in Table 1) was acquired from the Bacillus Genetic Stock Center (BGSC, http://www.bgsc.org) as a *B. subtilis* 168 background. The mutation was introduced into NCIB3610 through transformation of genomic DNA, and transformants were selected for on LB agar with kanamycin, generating strain LA247. The strain YC800 (*yvmC-cypX::tet*) was built using long-flanking homology PCR, which has been described in a previous study^66^. We used primers *yvmC*-P1, *yvmC*-P2, *cypX*-P1, and *cypX*-P2 (Table 1) to perform the PCR reactions. The final purified PCR product was transformed into PY79, and transformants were selected on specific antibiotic plates. Confirmation of deletion mutation was performed through PCR of the locus and Sanger sequencing.

To construct the plasmid pYC211, the DNA fragment containing the *gfp* gene was cut from pYC121 and cloned into the *Hind*III and *Bam*HI sites of pDG1730. The plasmids bearing *gfp* or *lacZ* reporters LA2, LA9, LA97, and LA163 (in DH5α) were designed using the following protocol. First, the promoter region of interest (or coding sequence) was PCR amplified from NCIB3610 using forward and reverse primers (listed in Table 1). The PCR product was purified after confirmation of successful amplification through agarose gel electrophoresis. Purified PCR and plasmid of interest were double digested separately using the same restriction enzymes (NEB), and ligated together using T4 DNA ligase (Invitrogen). The ligation reaction was transformed into chemically competent DH5α and, after overnight incubation of plates, colonies were picked. Promoter region insertion into the plasmid was confirmed through Sanger sequencing and DH5α strain was miniprepped to obtain enough plasmid mass for transformation into PY79. After selecting positive colonies for PY79 transformation through amylase production screening on starch agar plates, the genomic DNA of PY79 was isolated and transformed into NCIB3610, generating strains LA11, LA20, and LA174. Strains LA33 and LA224 were designed by transforming genomic DNA from PY79 containing the reporter of interest into a Δ*yvmC-cypX* background strain. Strains LA148 and LA262 were made by transforming genomic DNA from YQ141 into Δ*yvmC-cypX* and Δ*yvmA* backgrounds, respectively, and selecting for spectinomycin-resistant colonies and lack of amylase production.

Lastly, the dual reporter strain LA233 was constructed using two cloning steps. First, the LA2 plasmid was digested using restriction enzymes to remove the *gfp* coding sequence. Next, the *mkate2* gene was amplified from pYC251 using primers mkate2-F and mkate2-R, PCR purified, and cloned into the *gfp-*less plasmid to make LA72. LA72 was then digested using restriction enzymes and the DNA for the P_*yvmC*_*-mkate2* reporter sequence was gel purified and ligated into pDR183 plasmid (LA186). This plasmid was transformed into PY79 and the genomic DNA from a confirmed colony was transformed into LA174. Selection of colonies for both spectinomycin and erythromycin was performed, and the final construct generated was the dual reporter strain LA233.

### Biofilm assays

Prior to colony biofilm inoculation, cells were grown to exponential phase in LB broth. 2 μL of each cell culture were spotted onto LBGM 1.5% agar (w/v) and supplemented with 1 mM Isopropyl β-d-1-thiogalactopyranoside (IPTG)^45^. For pellicle biofilms, cells were grown to exponential phase in LB broth, and 3 μL of each culture was inoculated into 3 mL LBGM broth (1:1000 dilution) in a 12-well microtiter plate (Corning, NY, USA). Both colony and pellicle biofilm plates were incubated under static conditions at 30 °C. Pictures of colony biofilms were taken every 24 h using a Sony NEX-7 camera.

### Cell preparation and fluorescence microscopy

Pellicle biofilm cells were primarily broken down through vigorous pipetting. 3 mL of each cell suspension were transferred to 15 mL conical tubes (Fisher Scientific) and samples were mildly sonicated using a Sonifier^®^ cell disruptor (Heat Systems-Ultrasonics, Inc.) at the 1.5 output scale for 30 seconds to separate cells from the extracellular matrix. 1 mL of cell suspension was transferred to 1.5 mL microcentrifuge tubes (Fisher Scientific), spun down at 14,000 × *g* for 1 min, washed three times with phosphate-buffered saline (PBS), and resuspended in a final volume of 100 μL of PBS. Glass coverslips were previously treated with a sterile solution of 0.01% (v/v) poly-D-lysine before four microliters of each cell suspension were added onto glass microscope slides (Fisher Scientific). Images of cells were captured using a Leica DFC3000 G camera on a Leica AF6000 fluorescence microscope. Excitation and emission wavelengths used to visualize cells expressing GFP (and cells stained with the ROS dye) were 450–490 nm and 500–550 nm, respectively. The excitation and emission wavelengths used to visualize cells expressing mKate2 were 540–580 nm and 610–680 nm, respectively. By measuring wild-type cells without fluorescence, non-specific background fluorescence was defined. Cells grown under shaking conditions were prepared the same way as described above besides the sonication step. Each image obtained is representative of three biological replicates.

### Microplate growth curves

Each strain was grown overnight in 3 mL of LB broth with shaking at 200 rpm at 37 °C. The next day, cells were subcultured 1:100 in fresh LB broth at a final volume of 150 μL, and transferred into a 96-well tissue culture microplate (Corning, NY, USA). For cultures where IPTG was supplemented, the final concentration added to the medium was 1 mM. For the mitomycin C MIC test, a final concentration of 0.1, 0.25, 1, 2, 3, 4, or 5 μg/mL was added to the respective wells. For the H_2_O_2_ MIC test, a final concentration of 5, 10, 50, 100, 250, 500 or 1000 μM was added to the respective wells. Bulk culture growth by cell optical density (OD_600_ nm) was measured for 16 hours at 15-minute time point intervals in a BioTek plate reader under shaking conditions at 37 °C. Averages and standard deviations were calculated, and data were plotted as OD_600_ values vs time using Graphpad Prism 9. Three independent experiments were performed, and for each experiment, three biological replicates per strain were used. Statistical analysis was performed using Student’s *t* test on Graphpad Prism 9.

### β-Galactosidase activity assays

LBGM medium was used to cultivate pellicle biofilms of each strain as described above. At each time point, biofilms were softly sonicated, and either 1 mL or 100 μL of cell suspension were harvested in 2 mL microcentrifuge tubes (Fisher Scientific). Cells were pelleted by centrifugation at 14,000 × *g* for 1 minute, the supernatant discarded, and samples stored at −80 °C until use. Pellets were thawed at room temperature for 5 minutes, then resuspended in 1 mL of Z buffer (final concentrations: 200 mg/ml of lysozyme, 40 mM NaH_2_PO_4_, 60 mM Na_2_HPO_4_, 1 mM MgSO_4_, 10 mM KCl, and 38 mM β-mercaptoethanol). Samples were incubated at 30 °C for 15 minutes followed by addition of 200 μL of 4 mg/ml o-nitrophenyl-D-galactopyranoside (ONPG) to each tube to start the reaction. After a faint yellow color was observed in the tubes, 500 μL of 1 M Na_2_CO_3_ solution was added to stop the reaction, and the reaction time was recorded. To get rid of any cell debris, samples were spun down for 10 minutes at 5000 × *g*. The supernatants were transferred to 1 mL plastic cuvettes (Fisher Scientific) and absorbance readings at 420 and 550 nm were taken for each sample using a Bio-Rad SmartSpec 3000 spectrophotometer. The formula used to determine the β-galactosidase-specific activity was the following: [OD_420_ − 1.75(OD_550_)/(time × volume in mL × OD_600_)] × 1000. Results were plotted as bar graphs and statistical significance analyses were performed using Student *t* test on Graphpad Prism 9. Each experiment was performed three times, each with three biological triplicates per strain.

### Fluorescence quantification of single cells

The MicrobeJ plugin of the ImageJ software was used to quantify the fluorescence intensity for each cell from images obtained by fluorescence microscopy^55^. A minimum of 200 cells per strain and per treatment were measured. After quantifications, fluorescence values were plotted in a violin plot, where each dot represents a single cell, and the Student t-test was used to determine statistical significance amongst strains or treatments on Graphpad Prism 9.

### Determination of mutation frequency

In order to determine the mutation frequency between the wild type and the pulcherrimin mutant, we utilized a protocol based on a previously published study^58^. Wild-type and the pulcherrimin mutant were cultured separately in 250 mL flasks containing 50 mL LBGM broth supplemented with 0.2 mM FeCl_3_ for strong pigment production. After 24 hours of growth, aliquots of each culture were serially diluted and plated on LB agar for CFU quantification. For mutation frequency determination, a 1.5 mL aliquot of each culture was pelleted and resuspended in 150 μL of LB broth. The entire volume was plated onto LB agar plates supplemented with rifampicin to a final concentration of 5 μg/mL. Plates were incubated at 37 °C overnight and spontaneous rifampicin mutant colonies were counted the next day. Data visualization and statistical analysis were performed using Graphpad Prism 9.

### Challenging cell cultures with DNA-damaging agents

Thirty microliter (µL) of overnight cultures of the wild type and pulcherrimin mutant bearing the P_*recA*_*-gfp* reporter were subcultured in test tubes containing 3 mL of LB broth each (1:100 dilution). Tubes were placed in the shaker for 2 hours at 37 °C and 200 rpm. After that, experimental tubes were challenged with either mitomycin C or H_2_O_2_ at final concentrations of 0.25 µg/mL or 100 µM, respectively. Control tubes did not have either DNA-damaging molecule added. Tubes were placed back in the shaker for one more hour, then 1 mL of cells were harvested for each sample. Cells were washed with PBS three times and imaged under a fluorescence microscope. Fluorescence quantification was performed, data was plotted and statistical analysis was carried out using Student’s *t* test on Graphpad Prism 9. For each strain and treatment, a total of three biological replicates were tested.

### Killing assay using hydrogen peroxide

Cultures of the wild type and pulcherrimin mutant were grown overnight at 37 °C and 200 rpm in 50 mL LBGM supplemented with FeCl_3_ at the final concentration of 0.2 mM. On the next day, the volume of each culture was split into two new flasks. The first flask had H_2_O_2_ added at the final concentration of 10 mM. An equal volume of sterile water was added instead of H_2_O_2_ to the control flask. Flasks were placed back into the shaker for ten minutes, then 200 µL of cells were collected from each sample and serially diluted from 10^−1^ to 10^−10^ in sterile LB broth. Ten microliter (µL) of each serial dilution were plated on LB 1.5% agar, and plates were incubated overnight at 37 °C. Colony forming units (CFUs) were counted the next day. Percent cell survival was calculated by dividing the number of CFUs counted for the experimental flask by the number of CFUs in the control sample, multiplied by 100. Data were plotted as bar graphs and statistical significance analyses were performed using Student’s *t* test on Graphpad Prism 9. Each experiment was performed three times, each in biological triplicate per strain.

### Total ROS detection

Overnight cell cultures of the wild type and pulcherrimin mutant were inoculated in biological triplicate in 50 mL of LBGM supplemented with FeCl_3_ at the final concentration of 0.2 mM. On the next day, 1 mL of each culture was harvested, washed with PBS three times, and incubated with the Oxidative Stress Detection Reagent from the ROS-ID® Total ROS Detection Kit (Enzo Life Sciences) according to the manufacturer’s protocol. Cells were imaged using fluorescence microscopy, and fluorescence quantification, data representation on a violin plot, and statistical significance were performed as described above. The Oxidative Stress Detection Reagent is a cell-permeable, non-fluorescent molecule that directly reacts with a variety of reactive oxygen species, including hydrogen peroxide, peroxynitrite, and hydroxyl radicals, producing green fluorescence result indicative of ROS presence. Therefore, there is a positive correlation between the intracellular levels of ROS and the fluorescence intensity observed.

### Global transcriptome profiling by RNA-Seq

Pellicle biofilms of the wild type and pulcherrimin mutant were grown in LBGM as described above, and samples were harvested after 72 hours of development. Biofilms were softly sonicated, washed with PBS three times, measured for absorbance at OD_600_ nm, and a total of 10^9^ cells were pelleted through centrifugation. Cells were then incubated with RNAprotect® Bacteria Reagent (Qiagen) according to manufacturer’s protocol, pelleted again, snap frozen in liquid nitrogen, and stored at −80 °C until ready to ship. Genewiz (South Plainfileld, NJ) carried out the steps of RNA extraction, rRNA depletion, RNA fragmentation, quality control, library preparation, and sequencing through Illumina HiSeq 2x150bp. The raw data generated was submitted to the National Center for Biotechnology Information (NCBI, https://www.ncbi.nlm.nih.gov/bioproject/PRJNA630288). Sequence trimming and mapping were performed using Geneious Prime. The reference genome used to map the reads and to assess differential gene expression was NZ_CP020102 (*Bacillus subtilis* subsp. *subtilis* str. 168) from NCBI. The accession number for the reference sequence used for NCIB3610 plasmid pBS32 analysis was CP020103 from NCBI^50^.

### Measurements of pulcherrimin and pulcherriminic acid

Pulcherrimin was indirectly measured by converting it to pulcherriminic acid using 2 M NaOH solution. Briefly, 1 mL of each overnight LBGM shaking culture (with or without supplemented FeCl_3_) was spun down for one minute at 15,000 × *g* to pellet cells and insoluble pulcherrimin. The pellet was washed twice and resuspended in 1 mL 1× PBS. Following this, 300 µL of a 2 M NaOH stock solution was added and tubes were mixed by inversion ten times until all the pulcherrimin was converted from a reddish to a yellow color (pulcherriminic acid). Samples were spun down for 1 minute at 15,000 × *g* and 1 mL of supernatant was added to a cuvette. Following this, 410 nm measurements were performed using a Bio-Rad SmartSpec^TM^ 3000 spectrophotometer. OD600nm measurements of each overnight culture were also obtained for normalization of readings. Cultures of the pulcherrimin mutant grown overnight in LBGM at different FeCl_3_ concentrations were used as blanks for the assay. For the spent supernatant measurements of total pulcherriminic acid, each sample was first 10-fold diluted in 1× PBS, followed by 410 nm measurements. Spent supernatants of the pulcherrimin mutant with or without added FeCl_3_ were used as blanks for the assay.

### Reporting summary

Further information on research design is available in the Nature Research Reporting Summary linked to this article.

## Supplementary information

Reporting summary

Supplemental Figures

Supplemental Table 1

Supplemental Table 2

Supplemental Table 3

## Data Availability

The raw sequencing data generated in this study was submitted to the National Center for Biotechnology Information (NCBI, https://www.ncbi.nlm.nih.gov/bioproject/PRJNA630288). The authors declare that all other data supporting the findings of this study are available in this article and its Supplementary Information files, or from the corresponding author upon request.
