# Supplementary material for: Pulcherrimin protects Bacillus subtilis against oxidative stress during biofilm development
Source: NPJ Biofilms Microbiomes. 2023 Jul 19;9:50. doi: 10.1038/s41522-023-00418-z (PMC10356805; doi:10.1038/s41522-023-00418-z)
Supplement: Supplementary file 2 — Supplemental Figures [file 41522_2023_418_MOESM2_ESM.pdf]

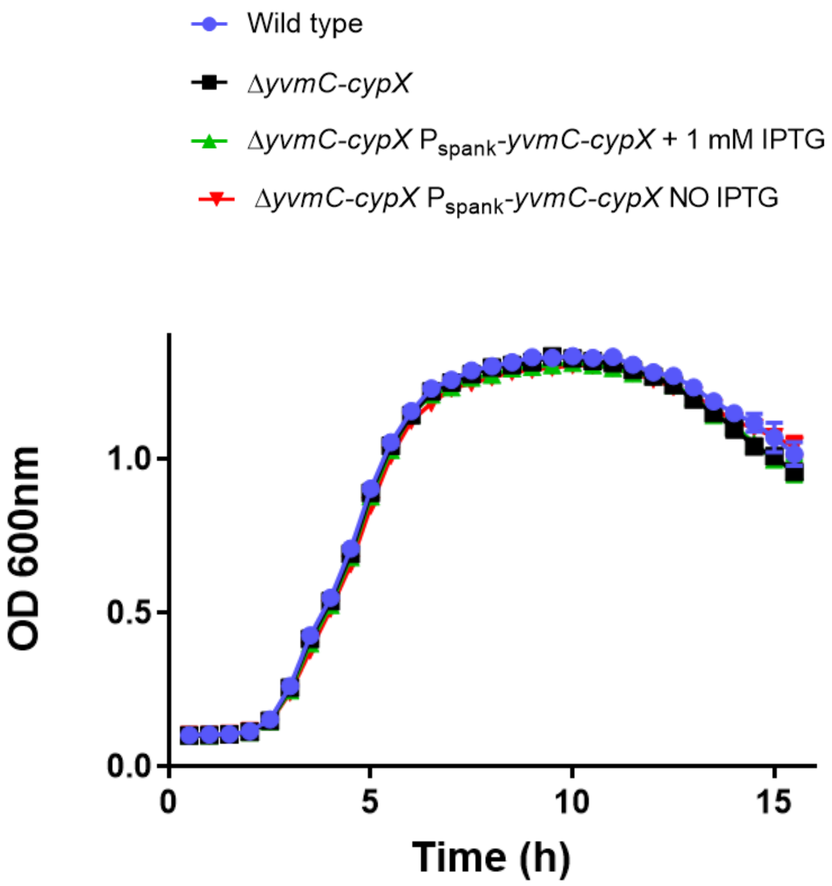

Supplemental Figure 1: Loss or overexpression of pulcherrimin biosynthesis genes does not affect cell growth of *B. subtilis.*
Growth of the wild type, pulcherrimin mutant and complementation strain in LB broth with or without IPTG supplementation at 1mM was accessed in a 96-well plate for 16 hours at 37°C. Experiment was performed with 8 technical replicates and average OD_{600} nm values were plotted using Graphpad Prism 9. Error bars correspond to standard deviation.

Figure S1

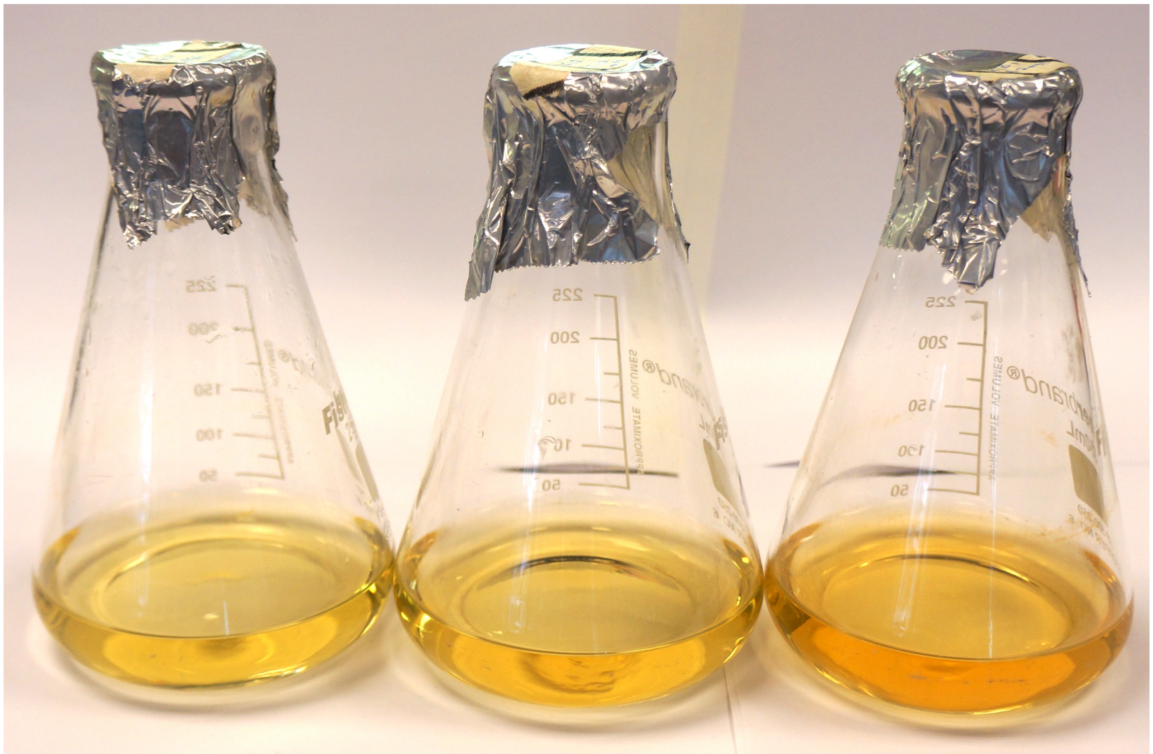

Regular LBGM **+0.05 mM $FeCl_3$** **+0.2 mM $FeCl_3$**

Supplemental Figure 2: $FeCl_3$ supplementation does not alter the color of LBGM broth. LBGM broth was prepared and aliquoted into 3 separate flasks. The first flask (left) did not have $FeCl_3$ added, the second flask (middle) had $FeCl_3$ added to a final concentration of 0.05 mM, and the third flask (right), to 0.2 mM. The broth color did not significantly change after $FeCl_3$ supplementation.

Figure S2

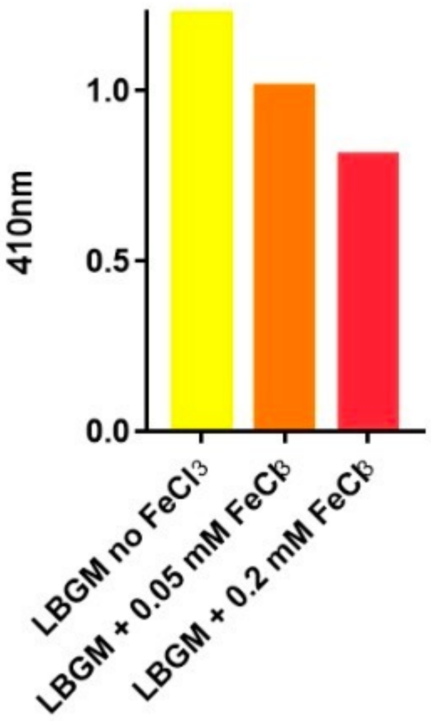

Supplemental Figure 3: The amount of pulcherriminic acid in the supernatant decreases with increasing $FeCl_3$ supplementation. Bar graphs represent values after quantification of total pulcherriminic acid from spent supernatants from overnight cultures of the wild-type strain with no $FeCl_3$ supplementation, 0.05 mM, and 0.2 mM $FeCl_3$. 410 nm values were plotted using Graphpad Prism 9.

Figure S3

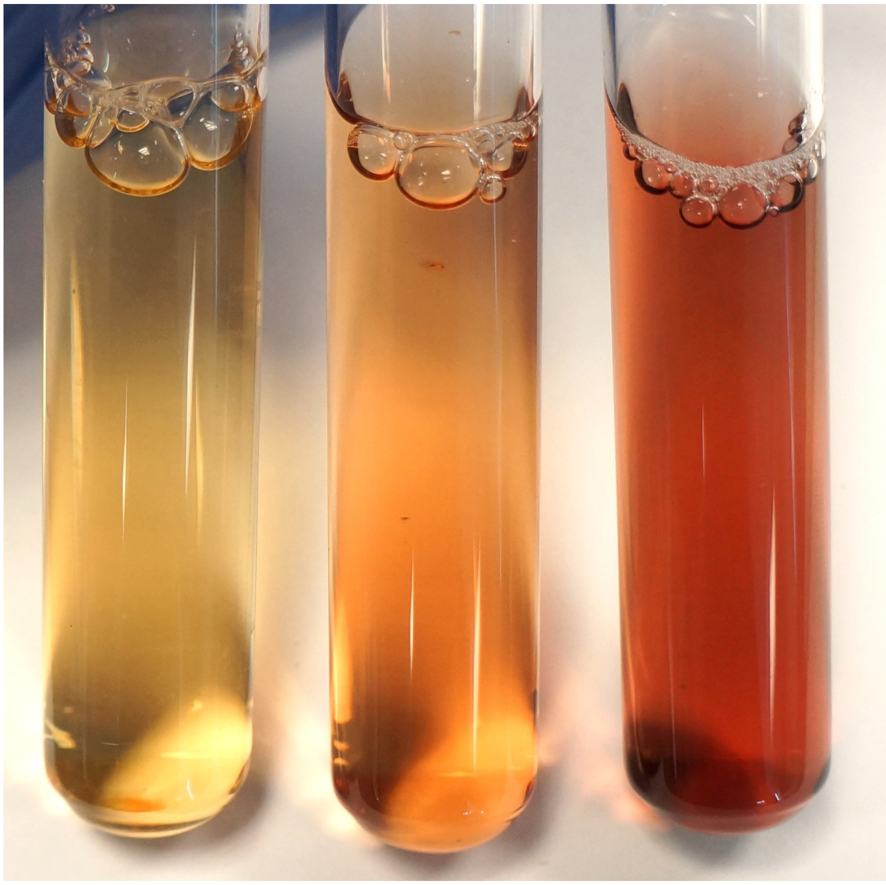

No addition +0.05 mM $FeCl_3$ +0.2 mM $FeCl_3$

Wild-type spent supernatants

Supplemental Figure 4: Addition of $FeCl_3$ to wild-type pellicle spent supernatants leads to conversion of pulcherriminic acid into the reddish-brown pigment pulcherrimin. Images obtained from a 3-days old wild-type pellicle filtered supernatant without $FeCl_3$ added (left), or supplemented with $FeCl_3$ to a final concentration of 0.05 mM (middle) or 0.2 mM (right). The supernatant turns from light yellow to a reddish-brown color (corresponding to pulcherrimin) once $FeCl_3$ is added.

Figure S4

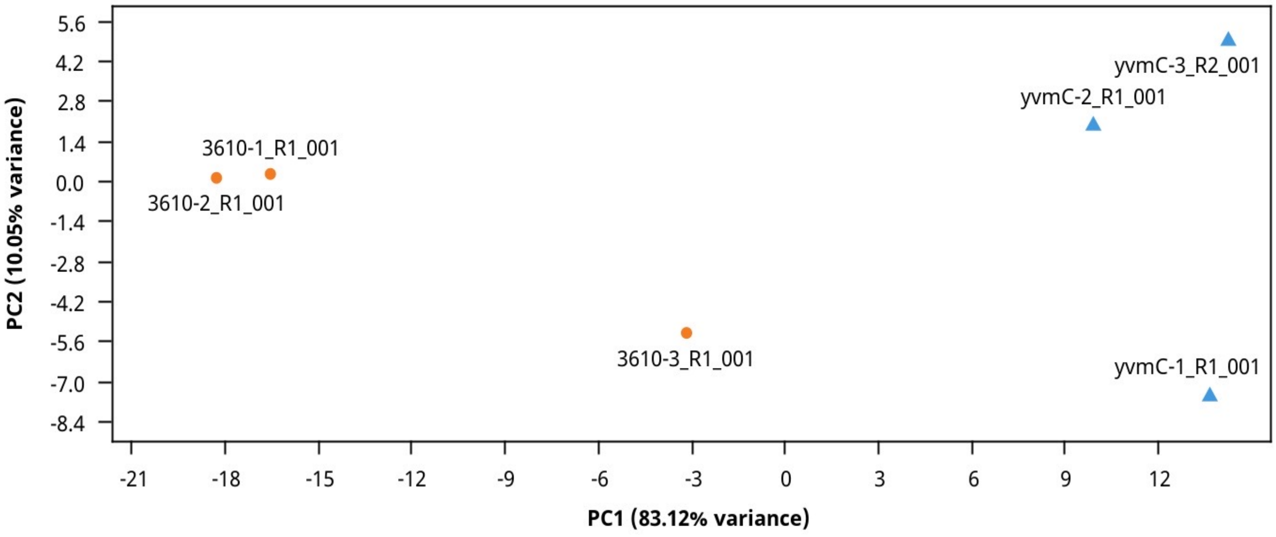

Supplemental Figure 5: Principal Component Analysis carried out with expression of 4,237 genes. Two principal components (PCs) with most of the variation are shown. PC1 (83.12%) separates the pulcherrimin mutant (Δ*ycmC-cypX*, blue triangles) from the wild type (3610, orange circles) RNA- Seq samples.

Figure S5

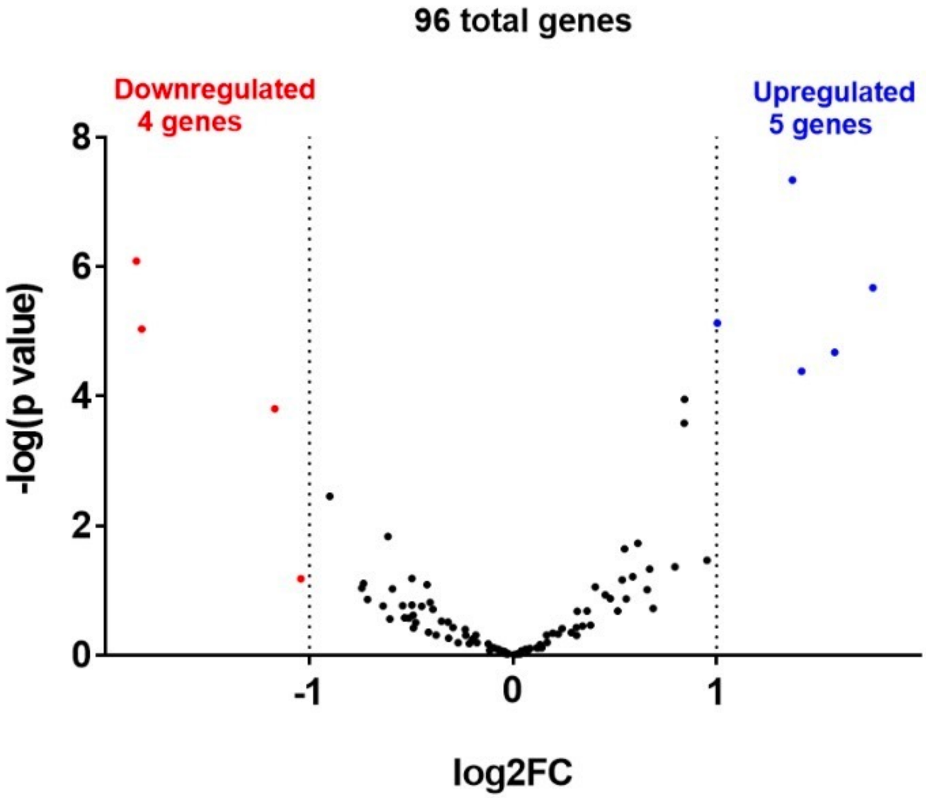

Supplemental Figure 6: Volcano plot generated after RNA-seq analysis using pBS32 plasmid from NCIB3610 as reference. Volcano plot depicting differentially expressed genes (blue and red dots) based on RNA-Seq analysis using pBS32 plasmid as reference (96 genes total). Downregulated genes with a log2FC of -1 or lower are depicted in red (4 total), and upregulated genes with a log2FC of 1 or higher are depicted in blue (5 total). Black dots represent genes not differentially expressed.

Figure S6
